# Molecular Heterogeneity of Core Binding Factor Acute Myeloid Leukemia (CBF-AML): Implications for Prognosis and for Treatment

**DOI:** 10.3390/hematolrep18050074

**Published:** 2026-10-08

**Authors:** Ugo Testa, Claudio Cerchione

**Affiliations:** 1 Department of Oncology, Istituto Superiore di Sanità, 00161 Rome, Italy; ugotesta@gmail.com; 2 IRCCS Istituto Romagnolo per lo Studio dei Tumori “Dino Amadori” (IRST), 47014 Meldole, Italy

**Keywords:** acute myeloid leukemia, core binding factor, CBF-AML, minimal residual disease, induction chemotherapy, consolidation therapy

## Abstract

**Background/Objectives:** Core binding factor acute myeloid leukemia (CBF-AML) is a subtype of AML characterized by specific genetic rearrangements, *RUNX1::RUNX1T1* and *CBFB::MYH11*, that disrupt the CBF complex, which is essential for normal blood cell development. Although CBF-AML is regarded as a favorable-risk AML, it remains a challenging disease with significant molecular heterogeneity and relapse rates around 40–50%. A better understanding of the molecular heterogeneity of CBF-AML and the development of induction regimens reducing the risk of relapse are required. **Methods**: A systematic search of the literature showed the development of studies aiming to define the genetic heterogeneity of CBF-AML and of clinical studies aiming to improve the outcomes of newly diagnosed or relapsed/refractory, fit and unfit patients. The aim of the present review is to critically analyze these studies. **Results** and **Conclusions**: Molecular studies have identified the presence of some genetic alterations, such as *KIT* or *FLT3-ITD* mutations that increase the risk of relapse. Incorporation of Gemtuzumab Ozogamicin (GO) into frontline induction chemotherapy improved the outcomes of fit CBF-AML patients. Treatments based on the BCL2 inhibitor Venetoclax have been developed for unfit CBF-AML patients and may improve their outcomes. Serial monitoring via quantitative polymerase chain reaction for the specific fusion transcripts (*RUNX1::RUNX1T1* or *CBFB::MYH11*) allows to define the minimal residua disease (MRD) and is crucial to monitor the effectiveness of treatments and to optimize the therapeutic strategy. The monitoring of MRD is also fundamental to define optimal strategies for consolidation strategy post-HSCT. These studies have contributed to identify CBF-AML patients who are at high risk of relapse and have to proceed to HSCT.

## 1. Introduction

Core binding factor acute myeloid leukemia (CBF-AML) is a distinct subtype of acute myeloid leukemia (AML) characterized by specific genetic rearrangements that involve the core binding factor complex, which is essential for normal blood cell development. The CBF complex is a heterodimeric transcription factor crucial for normal hematopoiesis (blood cell formation) and bone marrow development. It consists of a DNA-binding α-subunit (RUNX1, RUNX2 or RUNX3) and a non-DNA binding subunit (CBFβ) that stabilizes the complex [1].

RUNX1 and CBFβ are required for normal blood development, and the knockout of their genes in mice causes a block of fetal hematopoiesis [1]. RUNX and CBFβ are required for normal adult hematopoiesis; these genes are ubiquitously expressed in all hematopoietic lineages, including adult hematopoietic stem cells (HSCs) [1]. The function of these genes is also required for HSC maintenance and their multilineage differentiation. Thus, CBF complex is a master regulator in hematopoietic development, from the emergence of blood cells from embryonic hemogenic endothelium to the maintenance of adult hematopoiesis throughout an individual’s entire life.

CBFα (RUNX1/AML1) binds specifically to DNA at the consensus sequence 5′-YGYGGTY-3′, while CBFβ does not bind DNA directly but stabilizes the RUNX protein, prevents its degradation, and increases its affinity for DNA [1]. The main function of the CBF complex consists of the regulation of genes necessary for hematopoietic stem cell differentiation and skeletal development [1].

Disruption of the CBF complex via genetic translocations is a key driver genetic event in 10–15% of adult AML cases, defining a specific AML subtype with a relatively favorable prognosis. CBF-AML is defined by two primary cytogenetic abnormalities: t(8;21)(q22;q22), generating the *RUNX1::RUNX1T1 (AML1-ETO)* fusion gene, and *inv(16)(p13q22/:t(16;16)*, generating the *CBFB::MYH11* fusion gene. t(8;21) is often associated with FAB subtype M2, while inv(16) is associated with FAB M4eos (acute myelomonocytic leukemia with abnormal marrow eosinophils). In many studies these AMLs are treated together, but they represent two distinct entities.

A pivotal study by Appelbaum and coworkers defined the clinical spectrum of adult CBF-AML [2]. In this study some remarkable differences observed between inv(16) and t(8;21) AML were reported [2]. CBF-AML was characterized by a high rate of responses to standard induction chemotherapy, with a 10-year OS of 44% [2].

The incidence and survival of CBF-AML was evaluated in the context of a population-based study using the SEER (Surveillance, Epidemiology and End Results) database. The annual age-adjusted incidence rate was 0.11 per 100,000 persons; the incidence rates for the inv(16) and t(8;21) were comparable (0.05 and 0.07 per 100,000 persons) [3]. The population incidence of CBF-AML in the US general population increases with age, although the proportion of incident AML cases exhibiting CBF alterations decreases with age [3]. Thus, the population incidence of CBF-AML increased in the age cohorts of 15–44 years, 46–64 years, 65–74 years and 75–84 years from 0.06 to 0.13, to 0.25 and to 0.28, respectively [3]. The 3-year survival rate between years 2000 and 2010 were estimated to be 57.3% for inv(16) and 35.5% for t(8;21) [3].

## 2. Aim and Methodology

The aim of the present study is to review recent studies that have characterized the molecular heterogeneity of CBF-AML and have defined optimal strategies for the treatment of fit and unfit CBF-AML patients.

This study was based on a literature search of the PubMed database, using the terms acute myeloid leukemia, CBF-AML, t(8;21) AML, inv(16) AML, AML therapy, and KIT-mutant AML. The search period was expanded from 1990 to 2026. The inclusion criteria for the screened literature were limited to studies published in peer-reviewed publications in high-impact journals in hematology.

Furthermore, the Google search engine was used to find the link to major recent international hematology and oncology meetings, which were screened for congress abstracts related to CBF-AML.

## 3. Pathogenesis of CBF-AML

Translocation events determine an alteration of the CBF heterodimer DNA binding with an alternate DNA binding, which leads to a disruption of the normal transcription program, resulting in a block of the normal myeloid differentiation-maturation process [4]. This inhibitory activity on cell differentiation of the fusion protein AML1-ETO is exerted though a negative functional activity on transcription factors, such as PU.1, that are essential for normal myeloid cell differentiation [5].

The pathogenesis of CBF-AML requires the joint functional participation of fusion genes and additional mutant genes. Experimental studies of leukemogenesis in mice have shown that fusion genes are necessary but not sufficient to cause leukemia; particularly, the heterodimerization between AML1-ETO and CBFβ was shown to be required for leukemogenesis but not for inducing myeloproliferation [6].

Thus, the pathogenesis of CBF-AML can be described by a two-hit model, where the fusion genes act as class II mutations (impairing cell differentiation), which are necessary but not sufficient for leukemogenesis and require additional class II mutations (promoting myeloproliferation). The AML1-ETO fusion protein particularly acts as a dominant-negative inhibitor that binds to the DNA-binding domain of RUNX1, thus silencing the gene expression program required for normal myeloid differentiation; in addition, AML1-ETO forms a complex with normal RUNX1 inhibiting its function and recruits a complex of co-repressors, such as N-CoR, SMRT and HDACs, to silence RUNX1 target genes [4]. Therefore, the AML1-ETO fusion protein causes transcriptional and epigenetic deregulation by competing with WT RUNX1 for binding to DNA. A recent study provided direct evidence about the essential role of the AML1-ETO fusion protein within t(8;21) leukemic cells in promoting differentiation arrest; in fact, silencing *RUNX1::RUNX1T1* expression in primary t(8;21) leukemic blasts using small interfering RNA-loaded lipid nanoparticles induced a redirection of the transcriptional program toward myeloid differentiation, with depletion of myeloid stem cells and expansion of granulocytic and eosinophilic/mast cell-like populations [7]. The fusion CRFB-MYH11 protein binds RUNX1 and sequesters this protein in the cytoplasm, thus preventing its entry into the nucleus and causing differentiation–maturation arrest; this trapping mechanism is crucial for leukemogenesis, but also additional mechanisms of deregulation of transcription and signaling may contribute to the inhibitory effect exerted by the CBFB-MYH11 protein on myeloid differentiation [8,9]. Cooperating class I mutations are mutations required to promote the proliferation and survival of leukemic cells; examples of these mutations are given by *KIT*, *FLT3* (*FLT3-ITD* or *FLT3-TKD*) and *RAS* (*NRAS* or *KRAS*) mutations, frequently observed as co-mutations in inv(16) or t(8;21) leukemic cells.

The study of molecular signatures contributed to better understanding the pathogenetic processes underlying CBF-AML. CBF-AML is characterized by a distinct reactive oxygen species (ROS) signature that includes higher basal ROS production and increased mitochondrial stress compared to other AML subtypes. In fact, the study of redox balance in various AML subtypes showed that CBF-AML, inv(16) and t(8;21) AML showed significantly higher levels of ROS production than other AML subtypes and appeared highly sensitive to mitochondrial stress, seemingly reflecting the poor stability of mitochondrial permeability and a low mitochondrial threshold for apoptosis [10]. Despite high ROS production, CBF-AML showed fewer markers of chronic oxidative stress (such as lower malondialdehyde levels for inv(16) AML and lower oxidized glutathione for t(8;21) compared to other AML subtypes like *NPM1* or *FLT3-ITD*-mutant AML, thus supporting a peculiar redox regulation in CBF-AML [10].

A study on single base substitutional signatures in 20 pediatric AML cases analyzed by whole-genome sequencing showed in four t(8;21) AML cases the unique enrichment of the SBS18 signature, which is characterized by C>A transversions and is associated with damage caused by reactive oxygen species [11]. At the level of individual patients SBS18 contributed to 18–28% of the total mutational processes [11]. This initial study also showed that in addition to SBS18, SBS1 signature 1, associated with C>T transitions resulting from endogenous, spontaneous deamination of 5-methycytosine and increasing by age, is another frequent mutational signature observed in t(8;21) [11]. Frequent C>A transversions in pediatric t(8;21) AML were also observed by Bolouri et al., frequently associated with CBF-AML [12].

The AML1-ETO fusion protein seems to be involved in the genesis of this mutator phenotype through a direct effect on the promoter of the *OGG1* gene, a DNA glycosylase that excises oxidized guanines, leading to increased SBS18 [13].

Brandsma et al. subsequently also showed that SBS18 is common in childhood AML and particularly in cases of t(8;21) AML [14]. All these observations supported the conclusion that ROS could be involved in the genesis of childhood AML with t(8;21).

The evaluation of adult CBF-AML patients showed that the frequency of the SBS18 signature in adult t(8;21) and inv(16) AML was lower than that observed in pediatric cases; in adult t(;21) and inv(16) the most frequent mutational signature was SBS1 [15]. The lower frequency of SBS18 in adult patients could be related to the fact that SBS18 occurs early and did not increase with age, whereas other mutational signatures did [15]. The explorations of the contribution of the SBS18 signature to CBF-AML mutational processes was also confirmed by another study that extended the analysis of SBS18 signature to TCGA datasets, showing that in t(8;21) AML ROS-associated processes contribute not only mutations but also a significant oncogenic effect, supporting the development and maintenance of leukemia [16].

A recent whole-genome sequencing study carried out in 431 pediatric AML patients allowed an extensive analysis of mutational signatures using SigProfiler. SBS1 and SBS5, clock-like mutational signatures, were ubiquitously detected in different AML subtypes, and mutational burden was associated with age [17]. In AML with t(8;21) and inv(16), the reactive oxygen species mutational signature SBS18 contributed to a significant number of somatic mutations, with a clear association with age [17]. These observations indicated that SBS18 mutations accumulated at a consistent rate from birth to the onset of the disease and supported the in utero origin of t(8;21) translocations [18]. The frequency of SBS18 mutations was similar between clonal and subclonal mutations, suggesting that the mutational process mediated by ROS contributed to the entire process of clonal evolution of these AML subtypes [17].

## 4. Genetic Alterations in CBF-AML

As discussed above, experimental studies have clearly shown that fusion proteins AML1-ETO and CBFB-MYH11 block cell differentiation but are not sufficient to induce full leukemia development and require secondary mutations for leukemia development. Therefore, the identification of secondary mutations and other genetic alterations observed in inv(16) and t(8;21) cells is of fundamental importance.

Two key genetic studies in 2016 provided an extensive analysis of genetic variation contributing to CBF-AML development. Faber et al. reported a comprehensive analysis of 165 CBF-AML patients by whole-genome sequencing or by whole-exome sequencing (85 *RUNX1::RUNX1T1* and 80 *CBFB::MYH11*) (Figure 1). An average of 9.86 somatic mutations with functional consequences per case were observed; mutations were more frequent in AML1-ETO than in inv(16) AML (11.86 vs. 7.74, respectively) and in adult than in pediatric cases (12.56 vs. 6.55, respectively) [19]. A total of 66.1% of CBF-AML cases displayed mutations in cell signaling genes (activating mutations of *NRAS*, *KIT*, *FLT3*, *KRAS* and *PTPN11* and loss-of-function mutations of *NF1*). *NRAS* mutations were more common in inv(16) than in t(8;21); kit-exon 17 mutations were enriched in the t(8;21) cohort [19]. A striking difference between t(8;21) and inv(16) AML is given by loss-of-function mutations in epigenetic regulators, chromatin modifying genes *ASXL2*, *SETD2*, *KMT2D*, *KMT2C* and *CREBBP* more frequently mutated in t(8;21) than in inv(16) AML (44% vs. 4%, respectively); these mutations were detected in both pediatric and adult cases and were not associated with outcome. Another striking difference between t(8;21) and inv(16) AML involves a mutation in the cohesin complex (*SMC1A*, *SMC3* and *RAD21*) exclusively observed in t(8;21) AML; the mutation status of these genes has no effect on outcome [19]. Recurrent mutations in *DHX15*, a member of RNA helicases, were exclusively identified in the t(8;21) cohort [19].

In the t(8;21) AML, recurrent mutations were also observed in *CCND12* (10%), involved in hematopoietic cell proliferation, and *MGA* (8%), a negative regulator of *MYC* [19]. A subsequent study explored the occurrence of *CCND1* and *CCND2* gene mutations in t(8;21) AML and observed a total mutation frequency of 15% (*CCND1* 3% and *CCND2* 12%); *CCND2* mutations, all occurring around amino acid residue threonine 280, lead to increased phosphorylation of the retinoblastoma protein and increased proliferation [20]. *CCND2* mutations cooperate with AML1-ETO in inducing leukemia development in mouse models [21].

Loss of function mutations in the ZBTB7A gene, encoding transcription factor important for hematopoietic fate lineage decisions, are exclusively observed in t(8;21) AML (19% of cases) [22]; mutations of this gene include missense and truncating mutations resulting in loss of the C-terminal zinc-finger domain of *ZBTB7A*, with disruption of the transcription repressor potential and of the anti-proliferative effect [22].

In conclusion, this study showed that t(8;21) and inv(16) display a different spectrum of cooperating mutations.

In the second study, Duployez et al. reported the molecular characterization of 215 CBF-AML patients (109 with inv(16) and 106 with t(8;21)), and 85% of these patients had at least 1 mutation [23]. The mutation profile observed in this study was highly comparable to that reported by Faber et al. [19]. Particularly, mutations altering tyrosine kinase signaling were the most recurrent events in CBF-AML (80% in inv(16) and 65% in t(8;21)), with *KIT* and *NRAS* being the most frequent mutations; mutations in genes involved in chromatin modification, DNA methylation and cohesin were clearly more frequent in t(8;21() than in inv(16): 42% vs. 6%, 8% vs. 2% and 18% vs. 0%, respectively [23]. T(8;21) AML patients with mutations in kinase signaling plus chromatin modifiers or cohesin members had the highest risk of relapse following induction chemotherapy [23].

These studies have suggested that inv(16) AML is largely driven by alterations in RTK/Ras signaling, in contrast to t(8;21), which is more complex and characterized by multiple interactions of different genes within a variety of functional pathways.

In addition to the primary cytogenetic abnormality related to the CBF translocation, CBF-AML may also harbor secondary cytogenetic alterations. Han and coworkers have reported a comprehensive analysis of secondary cytogenetic abnormalities in a large cohort of 537 CBF-AML harboring inv(16) or t(8;21) [24] (Figure 2). The results of this extensive analysis showed that trisomies of chromosomes 8, 21 or 22 were more frequent in patients with inv(16) than in those with t(8;21): 16% vs. 7%, 6% vs. 0%, and 17% vs. 0%, respectively [24]. However, del(9q) and loss of a sex chromosome were more frequent in patients with t(8;21) compared to those with inv(16): 15% vs. 0.4% for del(9q), 37% vs. 0% for loss of X chromosomes in females, and 44% vs. 5% for loss of chromosome Y in males [24]. Hyperdiploidy was more frequent in patients, with inv(16) being more frequent in patients with t(8;21) (37% vs. 3%) [24]. In multivariate analysis, in inv(16) patients, trisomy 8 was associated with better survival, while the rest of the cytogenetic abnormalities were associated with reduced OS [24]. In patients with t(8;21() hypodiploidy was associated with improved DFS; hyperdiploidy and del(9q) were associated with improved OS [24].

Jahn and coworkers explored the genomic heterogeneity in 350 adult CBF-AML patients (160 inv(16) and 190 t(8;21)). The genetic analysis of these patients by targeted sequencing confirmed the results previously reported for mutational profile and for cytogenetic abnormalities. In addition, the study provided an analysis of the clonal architecture of CBF-AML. In the t(8;21) AML, mutations in genes involved in DNA methylation or encoding transcription factors usually occurred earlier, followed by genes involved in chromatin modification and genes belonging to the cohesin complex and finally by gene mutations in genes involved in RTK/RAS signaling pathways [25]. A similar clonal hierarchy, although less complex, was observed in inv(16) AML [25].

Another study based on the genomic characterization of 331 t(8;21) AML patients was in line with the previous study showing that epigenetic regulator mutations for their high mutant levels, pairwise precedence, and stability at relapse are early events preceding signaling mutations; in contrast, signaling mutations are relatively late events and impact patient outcome [26]. The variant allele frequency (VAF) of cohesin complex mutations was variable, with some mutations (*RAD21* and STAG2) representing late events and other gene mutations (*SMC1A*, SMC3) early events [26]. Whole-exome sequencing analysis on 19 paired diagnosis, remission and relapse showed that some mutations exhibit signs of clonal evolution; according to the mutation profiles at relapse two groups of patients were identified, one genetically stable (13/19) and the other unstable (6/19) [26]. In the genetically stable group ≥40% of stable mutations (mutations present at diagnosis and at relapse) are observed, while in the genetically unstable group ≥60% of gained/lost mutations are observed. The t(8;21) fusion was detected in all patients at diagnosis and relapse, suggesting common ancestral clones. Although the clonal evolution seemed to follow a patient-specific pattern, some patterns of genetic evolution seemed to be gene-specific: mutations in epigenetic regulators and cell-cycle regulators are usually stable and never acquired just at relapse, supporting their role in leukemic initiation; mutations in *RAS/RTK* signaling pathway genes, cohesin complex genes and splicing machinery genes are variable in their clonal evolution, being stable, lost or acquired at relapse [26].

At the molecular level, inv(16) translocations result in the juxtaposition of the myosin, heavy chain 11, smooth muscle gene (*MYH11*) at 16p13 and the *CBFB* gene at 16q22, with generation of the *CBFB::MYH11* fusion gene. The breakpoints within *CBFB* and *MYH11* are variable, generating more than *CBFB::MHY11* fusion transcript variants of different size; in adult inv(16) patients more than 85% of the fusions are type A and 15% of type non-A [27]. Remarkable differences exist between type A and non-type A patients in that non-type A patients do not have *KIT* mutations (compared to 27% in type A patients), have lower white blood counts, have more frequent trisomies of chromosomes 8 and 21 and have less frequent trisomy 22 [27]. Among type A patients, the presence of *KIT* mutations conferred adverse prognosis [27]. Similar observations were made in pediatric patients with type A *CBFB::MYH11* AML associated with 42% of *KIT* mutations compared to 7% in non-type A *CBFB::MYH11* AML [28].

Using integrative analysis of immunophenotype, RNA sequencing and single-cell RNA sequencing, Jiang et al. identified in t(8;21) AML patients three populations of leukemic cells exhibiting a progressive differentiation level: CD34^+^/CD117^dim^ quiescent blasts corresponding to early myeloid cells resembling granulo-monocytic progenitors; CD34^+^/CD117^bright^ proliferating blasts corresponding at early myeloid precursors; and leukemic blasts corresponding to myeloid cells with partial maturation [29]. T(8;21) AML patients were heterogeneous for the frequency of CD34^+^/CD117^dim^ cells, and patients displaying a higher frequency of these cells have a significantly lower RFS and OS compared to those with a lower proportion of these cells [29]. The mutational profiles of t(;21) AML with a high and low frequency of CD34^+^/CD117^dim^ cells was similar, except for a lower frequency of *NRAS* mutations and a higher frequency of *SMC1A* mutations in the former ones compared to the latter ones [29]. The two CD34^+^/CD117^dim^ low and high populations were distinguished into two subgroups according to *KIT* mutational status; the subgroup with a high frequency of CD34^+^/CD117^dim^ and with *KIT* mutations was associated with a particularly poor prognosis [29].

The study of immunophenotypic features of t(8;21) AML showed a consistent heterogeneity in that part of the patients can be classified with a primitive differentiation profile (42.3% of total) and another part with a monocytic differentiation profile (35.2% of total); the co-mutation profile of these two groups of patients was comparable [30]. However, the stage of differentiation and some gene mutations had a consistent impact on clinical outcomes. Thus, the monocytic group had a significantly shorter 2-year OS and a higher cumulative incidence of relapse compared to the primitive group; this difference was observed for both patients treated with intensive chemotherapy (IC) alone or with allo-HSCT after IC [29]. In both monocytic and primitive groups of patients the presence of *KIT* mutations negatively impacted the response to therapy [30].

## 5. Mutational Landscape at Relapse

While considered favorable risk, CBL-AML relapse occurs in about 30–50% of patients, indicating that induction chemotherapy was unable to eradicate all leukemic clones. Relapse rates were estimated around 35% for t(8;21) and 40% for inv(16). Molecular relapse precedes morphological relapse, and assessment of minimal residual disease (MRD) represents a fundamental tool to predict response to treatment and risk of relapse.

The prognosis of relapsed patients is poorer than that of patients at diagnosis, and the treatment of these patients often requires allogeneic HSCT.

Few studies have explored the mutational profile of t(8;21) and inv(16) AML patients at relapse. Sood et al. reported the analysis of 10 CBF-AML patients at diagnosis and at relapse by whole-exome sequencing; the majority of mutant genes were shared between diagnosis and relapse, a minority was observed only at diagnosis, and other mutant genes were specific for relapse. Concerning the most common co-mutations observed in CBF-AML, five patients had *FLT3-ITD* mutations at both diagnosis and relapse and one additional patient acquired a *FLT3* mutation at relapse; four patients had *KIT* mutations at both diagnosis and relapse; and *NRAS* mutations were present in three patients at diagnosis and only in one of these patients at relapse [31]. The deduced clonal evolution through integrated analysis of the mutational profile and the copy number variation supported two different mechanisms of relapse: (i) a subclone present in the leukemia at diagnosis survived therapy and reemerged after accumulating additional mutations; (ii) a preleukemic clone survived leukemia therapy, acquired additional mutations during remission, and gave rise to relapse leukemia [31] (Figure 3). Both these mechanisms imply the existence of a leukemic clone resistant to therapy. This study also showed that in all relapsing patients *RUNX1::RUNX1T1* and *CBFB::MYH11* fusion genes were present both at diagnosis and relapse, mutations in one or more of the known AML driver genes tend to be present at both diagnosis and relapse, and most of the new mutations occurring at relapse involve genes not previously linked to AML [31].

Dolnik reported whole-exome sequencing of 47 CBF-AML at diagnosis, remission and relapse. In t(8;21) AML a median of 3.5 mutations were present only at diagnosis, 11.6 persisted from diagnosis to relapse and 4.0 mutations gained at relapse were observed; in inv(16) AML a median of 2.0 mutations were present only at diagnosis, six mutations persisted from diagnosis to relapse, and 2.5 mutations gained at relapse were observed [32]. Mutations in relapsed t(8;21) AML patients often affected genes involved in PI3K-AKT and in cell cycle regulation pathways. In the inv(16) AML cases mutations acquired in relapsed samples mostly involved the MAPK signaling pathway and non-recurrent gene mutations in genes involved in RNA metabolism [32].

Hollein et al. analyzed by targeted sequencing 21 t(8;21) AML patients who reached molecular remission following intensive chemotherapy and then relapsed [33]. At diagnosis, patents who relapsed following molecular remission had a higher burden of co-mutated genes than patients that did not relapse [33].

## 6. Prognostic Relevance of Secondary Gene Alterations in CBF-AML

As discussed above, some gene alterations such as *KIT*, *FLT3*, *NRAS*, *ZBTB4*, *CCND2*, and *JAK2* mutations, currently observed in CBF-AML, play a relevant role in leukemia development in cooperation with the two specific fusion genes (Table 1). Consequently, it is expected that some of the mutations may affect the outcomes of CBF-AML patients. Therefore, several studies have explored the clinical impact of the secondary genetic alterations observed in CBF-AML (Table 1). Some studies were based on the analysis of clinically and molecularly characterized large cohorts of CBF-AML patients, while other studies focused on the analysis of single gene mutations.

Opatz and coworkers explored the clinical mutatome in a cohort of 292 CBF-AML patients characterized for genetic alterations by whole-exome and targeted gene sequencing and for clinical features and response to treatment. This analysis confirmed the fundamental differences observed between the mutational profile of inv(16) and t(8;21) AML [34]. In this study, the OS observed for t(8;21) AML was similar to that found for inv(16) AML; in both these groups of patients, patients aged above 60 years had a shorter OS than younger patients [34]. Multivariate Cox regression analysis showed that *KITD816*, *DHX15* and *SMC1A* mutations were independent prognostic factors. Particularly, mutations in *DHX15* and *SMC1A* correlated with shorter OS in t(8;21), while point mutations in *KIT* at position D816 correlated with reduced RFS in t(8;21) AML and the mutational burden of *KIT816* mutations was not prognostically relevant [34]. Alterations of genes encoding components of the cohesin complex together resulted in significantly shorter OS but not RFS in univariate analysis [34]. Secondary chromosomal alterations may also influence the outcomes of CBF-AML patients. In fact, *CBFB::MYH11*-rearranged patients with trisomy 8 or trisomy 22 had a tendency toward longer RFS than patients without these trisomies [34]. Loss of the Y chromosome in male patients correlated with improved RFS in univariate analysis in t(8;21) AML patients [34].

*DHX15* mutations were observed in 4.6% of t(8;21) patients in [24]. The *DHX15* gene is part of the splicing machinery and missense mutations at R222, observed in t(8;21) AML, resulting in increased numbers of alternate splicing events. An experimental study showed that *DHX15* mutations resulted in lower levels of *DHX15*, causing an increased production of the spliced AML-ETOa splice variant (containing a stop codon between exon 8 and exon 9 of ETO and generating a truncated AML-ETOa variant lacking ETO domains NHR 3–4) that alone causes leukemia in mice [35].

Concerning *SMC1A* mutations, it is important to note that these mutations were found mostly with high allelic frequency, likely representing the predominant clone [34].

In this study, *CSF3R* mutations were observed in a minority of t(8;21) AML patients; these mutations were shown to cooperate with AML1-ETO in experimental models [36] but are not associated with a negative outcome in t(8;21) patients [37].

Yiang and coworkers retrospectively analyzed 536 CBF-AML patients aged 60 years or younger; they characterized them for clinical response and evaluated them for mutational profile [28]. For patients with t(8;21), the presence of *KIT* and *TET2* mutations adversely correlated with OS and RFS with a hazard ratio of 2.81 and 3.80 for OS and 2.86 and 3.57 for RFS, respectively [28]. In contrast, in these patients, *NRAS* mutations were indicative of better OS and RFS, with an HR of 0.31 for OS and 0.38 for RFS [38]. In contrast, in inv(16) patients, these genetic alterations failed to have a significant impact on the survival of patients. Concerning chromosomal abnormalities, in t(8;21) AML patients, neither sex chromosome loss nor chromosome 9q deletion significantly impacted survival outcomes. In inv(16) patients, trisomy showed a trend toward better RFS but not OS, while trisomy 22 did not significantly influence survival outcomes [38]. Importantly, multivariate analysis incorporating clinical parameters, induction regimens and mutational profiles confirmed that in t(8;21) but not in inv(16), patients’ mutations in *KIT*, *TET2* and *NRAS* served as independent prognostic factors for [38].

Yu and coworkers have explored the impact of genetic abnormalities through the retrospective analysis of 346 CBF-AML (211 with t(8;21) and 135 with inv(16)) fully characterized at clinical and genomic levels [39]. In the t(8;21) AML group, the 3-year EFS, RFS and OS rates were 38%, 66% and 77%, respectively; in the inv(16) AML group the 3-year EFS, RFS and OS rates were 38%, 69% and 83%, respectively [39]. In the t(8;21) AML, multivariate analyses showed that *KDM6A* mutations were significantly associated with poor OS and RFS; *FLT3-TKD* mutations with poor OS; and *KIT* mutations (VAF > 25%) with poor RFS; *ASXL1* mutations with favorable EFS and OD [29]. In the inv(16) cohort, multivariate analyses provided evidence that a high mutation burden was associated with lower OS and *FLT3-ITD* mutations with inferior OS [39]. In addition, in the t(8;21) cohort, increasing age, not intensive chemotherapy, and high MRD levels predicted poor outcomes [39].

A recent study showed that *KDM6A* mutations are rare in AML, with exception of t(8;21) AML where they are recurrent; the presence of *KDM6A* mutations in t(8;21) AML was associated with increased relapse risk [40].

A recent study by Vasseur et al. explored the prognostic relevance of secondary genetic alterations when accounting for MRD in a large cohort of 656 CBF-AML patients (46% t(8;21) and 54% inv(16) AML) in CR after one induction course of intensive chemotherapy [41]. OS was similar in the two CBF subtypes (3-year OS 79% for t(8;21) and 81% for inv(16)); 3-year cumulative incidence of relapse (CIR) was 36% in t(8;21) and 42% in inv(16) [41]. BM MRD with a threshold of 10^−3^ after the first induction cycle was the most informative early MRD biomarker in t(8;21) AML patients; PB MRD with a threshold of 10^−4^ after the first induction cycle was the most informative early MRD biomarker in inv(16) AMLs [41]. In t(8;21) AML patients, 3-year CIR with BM-MRD1 < 10^−3^ was 21% and 47% for BM-MRD1 ≥ 10^−3^; in inv(16) AML patients with PB-MRD1 evaluation, 3-year CIR was 32% for PB-MRD1 < 10^−4^ and 49) for PB-MRD1 ≥ 10^−4^ [32]. In t(8;21) AML, *KIT* mutations increased the risk of relapse, but poorer prognosis was only associated with *KIT-TKD* mutations and not with mutations in other domains of the receptor: at 3 years, CIR was 61% with *KIT-TKD*, 30% with other *KIT* mutations and 32% without *KIT* mutations [32]. In *KIT-TKD*-mutated patients with available BM-MRD1 evaluation (65% of these patients had BM-MRD1 > 10^−3^) risk of relapse was higher regardless of MRD [41]. Overall survival was 67% for *KIT-TKD/BM-MRD1* < 10^−3^ and 59% for *KIT-TKD/BM-MRD1* ≥ 10^−3^ [41]. In inv(16) AML patients, the presence of any *FLT3* mutation was associated with a higher risk of relapse; this prognostic value was mostly related to *FLT3-ITD* and less to other *FLT3* mutations: 3-year CIR was 69% for patients with *FLT3-ITD*, 51% with other *FLT3* mutations and 35% without *FLT3* mutations [41]. In these patients, *KIT-TKD* nor other *KIT* mutations impacted the risk of relapse. In the inv(16) cohort, 3-year CIR was 26% in *FLT3-ITD^neg^/PB-MRD1* < 10^−4^, 80% in the *FLT3-ITD^pos^* patients regardless of PB-MRD1 and 52% in the *FLT3-ITD^neg^/FLT3-MRD1* ≥ 10^−4^ [41].

### 6.1. Prognostic Relevance of KIT Mutations

As discussed above, *KIT* mutations are recurrent in CBF-AML, being observed in about 20–40% of cases, with higher frequency often noted in adult patients compared to pediatric cases [10,14]. The *KIT* gene, located at chromosome band 4q11-12, encodes a 145 KD transmembrane glycoprotein, which is a member of the type 3 receptor tyrosine kinase family. The Kit protein is a glycoprotein of 976 amino acids, organized into several distinct domains: an extracellular domain consisting of five immunoglobulin-like domains that bind to the ligand (KIT Ligand, KL); a transmembrane segment consisting of a 23 amino acid hydrophobic segment that anchors the protein to the cell membrane; a juxtamembrane domain involved in the inhibition of receptor activity in the inactive state; and an intracellular tyrosine kinase domain (TKD) containing two tyrosine kinase lobes with an inserted sequence of about 80 amino acids, responsible for receptor autophosphorylation and activation. After KL binding, the KIT receptor dimerizes, becomes phosphorylated, and activates downstream signaling pathways involved in proliferation, differentiation and survival. Particularly, following binding with its ligand, KIT forms a dimer that activates its tyrosine kinase activity, leading to autophosphorylation at tyrosine residues; activated KIT acts as a docking site for signaling molecules stimulating cell growth, proliferation, survival and differentiation. KIT function is essential for the survival and development of specific stem cells and tissues, particularly hematopoietic stem cells. KIT signaling is regulated though a process implying rapid receptor ubiquitination and degradation via lysosomes following activation.

KIT mutations often affect receptor domains critical to RTK activity and may result in ligand-independent constitutive receptor activation. In CBF-AML, *KIT* mutations most frequently cluster in two receptor regions located within exon 17 encoding the KIT activation loop (A-loop) in the receptor tyrosine kinase domain (KIT-TKD mutations) and in exon 8, encoding an evolutionary conserved region (IgG domain) in the extracellular portion of the KIT receptor. Mutations in the IgG domain (exon 8) have no functional consequences and no prognostic impact; in contrast, mutations in the TKD (exon 17) resulted in aberrant KIT phosphorylation and were associated with inferior clinical outcomes [42]. Importantly, *KIT-TKD* mutations confer sensitivity to KIT-directed tyrosine kinase inhibitors [42].

In a group of adult CBF-AML patients it was reported among inv(16) patients a frequency of 29.5% of *KIT* mutations, 16% with *KIT-Exon 17* and 13% with *KIT-Exon 8* mutations; in t(8;21) patients, there was a frequency of 22% *KIT* mutations, with 18% having *KIT-Exon 17* mutations and 4% having *KIT-Exon 8* mutations [43]. In a group of pediatric patients, *KIT* mutations were detected in 31% of patients, with 35% involving only exon 8, 51% only exon 17, 10% both exons and 5% alternative exons [44].

*KIT* mutations are heterogeneous; thus, most *KIT* mutations are small deletions and/or insertions in exon 8, leading to the replacement of codons 417–419 or point mutations in *KIT* exon 17. Most exon 17 mutations involved codons D816 and N822 in the activation loop of the TKD [23]. Furthermore, Duployez et al. reported a higher rate of *KIT*-exon 17 mutations in t(8;21) than in inv(16) AML (25% vs. 15% of all patients [23].

Qin et al. reported an extensive characterization of *KIT* mutation types in a group of 114 t(8;21) AML patients with *KIT* mutations; 80.7% had sole *KIT*-exon 17 mutations, 9.6% sole *KIT*-exon 8 mutations and 9.6% compound *KIT*-exon 8 and -exon 17 mutations [45]. Among exon 17 mutations the most recurrent are D816V (3.5%), N822K (24.6%), D816Y (8.8%) and D816H (7%) [43]. *KIT*-exon 17 but not *KIT*-exon 8 mutations negatively affected outcomes of these patients; *KIT^D816^* mutations but not *KIT^N822^* mutations reduced RFS and OS of t(8;21) AML patients [45].

Other studies have shown that *KIT^D816^* mutations in t(8;21) AML are associated with reduced EFS and OS [46]. Furthermore, both t(8;21) and inv(16) AMLs *KIT^D816^* mutations are associated with poorer prognosis than other *KIT* gene mutations, including also *KIT^N822^* mutations [47].

Patients with *KIT* mutations are heterogeneous for the type of *KIT* mutation and also for the level of expression of KIT-mutant protein related to variation in allele frequency. A first study by Allan et al. showed that *KIT*-mutant levels are highly variable in CBF-AML; *KIT* mutations were associated with a higher CIR, but in multivariate analysis this was only significant for cases with a higher mutant level of 25% or greater [48]. Jang et al. evaluated the level of *KIT*-exon 17 mutant and identified a cutoff level to distinguish high expression from low expression in a group of CBF-AML patients; they reached the conclusion that cases with high *KIT*-mutant expression are associated with lower RFS and OS [49].

Duployez et al. showed that *KIT* mutations were associated with a significantly higher CIR for t(8;21) AML patients with a mutant allelic ratio of 35% or greater (*KIT* ≥ 35%) [23]. *KIT* ≥ 35% patients have a CIR markedly higher than *KIT* < 35% patients (69.4% vs. 30.7%, respectively) [23].

Importantly, the HARMONY platform reported the results of 520 AML patients with t(8;21) aged 16–70 years who received IC ± GO [49]. Both univariate and multivariate analyses (VAF) ≥ 25% (KIT high) were associated with a high risk of AML relapse (2-year CIR 50% vs. 26% for KIT high vs. KIT low (VAF < 25%) or KIT-WT) [50].

A consistent number of studies provided evidence that *KIT* mutations, particularly exon 17 mutations in AML with t(8;21), are associated with inferior outcomes [23,25,26,43,47]. In this context, a study by Ishikawa and coworkers was particularly relevant, which characterized for the *KIT* mutational profile 199 newly diagnosed CBF-AML patients [51]. In the whole leukemic CBF-AML group of patients the RFS in *KIT*-mutated patients was inferior to that observed in unmutated patients. Based on subgroup analysis, *KIT* mutations had a prognostic impact in patients with t(8;21) but not in those with inv(16), and only exon 17 mutations had a significant prognostic impact [51].

The combined evaluation of *KIT* mutational status and MRD status through the evaluation of AML1-ETO transcript levels improved the risk stratification of t(8;21) AML patients. Qin et al. reported the evaluation of 287 t(8;21) AML patients, 120 with *KIT* mutations and analyzed for the *KIT* mutation subtype [52]. Following mutation subtype, t(8;21) patients were subdivided into three subgroups: *KIT^D816/D820^*, *KIT^N822^* and *KIT-WT*. *KIT^D816/D820^* mutations had a significantly higher CIR than *KIT^N822^* and *KIT-WT* patients (3-year CIR 58.9% vs. 22.6% and 19.0%, respectively) [52]. MRD was evaluated at CR, after first (MRD1) and second consolidation (MRD2); 2-log reduction and 3-log reduction at MRD1 and MRD2, respectively, were selected as cutoff values to distinguish MRD^high^ and MRD^low^ patients [52]. The combined evaluation of the *KIT* mutational profile and MRD status allowed distinguishing three groups of patients: high risk (*KIT^D816/D820^* patients with MRD^high^), intermediate risk (*KIT^D816/D820^* patients with MRD^low^, *KIT^N822^*, *KIT^Exon8^* and *KIT-WT* with MRD^high^); and low risk (*KIT^Exon8^* and *KIT-WT* patients with MRD^low^) [52].

Duan et al. confirmed these findings and showed the identification of high-risk CBF-AML patients with *KIT^D816/D820^* mutations and MRD > 0.1% after the second consolidation [53].

It is of interest that in t(8;21), AML overexpression of KIT mRNA levels was reported both in *KIT*-mutant and *KIT*-WT patients [53]. In a cohort of t(8;21) patients it was observed that in about 50% of *KIT*-mutant patients and 35% of *KIT*-WT patients, KIT mRNA overexpression was observed [54]. In the whole population of t(8;21) AML patients, *KIT^high^* expression was associated with reduced EFS and OS, independently of *KIT* mutations [55]. The AML1-ETO fusion protein transactivates KIT expression through transcriptional mechanisms [55].

A complex karyotype (CK) defined by the presence of t(8;21) or inv(16) plus two or more additional secondary cytogenetic abnormalities was observed in 17% of inv(16) and 25% of t(8;21) [24]. A minority of *KIT*-mutant t(8;21) AML patients harbor a CK and represent a subgroup of high-risk patients [56]. T(8;21) AML patients have been stratified into three subgroups, low risk (CK^−^/*KIT*^−^), intermediate risk (CK^+^ or *KIT*^+^) and high risk (CK^+^/*KIT*^+^), with 5-year OS rates of 72.8%, 58.3% and 41.6%, respectively [56].

Recent meta-analyses on the literature data on the prognostic implications of secondary mutational and cytogenetic alterations in CBF-AML have concordantly shown the negative impact of *KIT* mutations in t(8;21) but not in Inv(16) AML [57,58].

### 6.2. Prognostic Relevance of FLT3 Mutations

*FLT3* mutations are less frequent than *KIT* mutations in CBF-AML, being estimated in the range of 15–20% positivity. *FLT3* mutations are observed in 10–15% of t(8;21) AML, with *FLT3-ITD* mutations being more frequent than *FLT3-TKD* mutations; *FLT3* mutations are more common in inv(16) AML than in t(8;21) AML. *FLT3-TKD* mutations are often more common in inv(16) compared to t(8;21) AML. In fact, the analysis of 21 CBF-AML studies involving a total of 1053 patients showed in inv(16) patients a mutational frequency of *FLT3-ITD* and *FLT3-TKD* of 4.9% and 13.3, respectively, compared to 7.1% and 3.6%, respectively, in t(8;21) AML [59]. In a large screening on 1151 de novo AML patients with *FLT3* mutations Chong et al. confirmed that *FLT3-TKD* mutations are markedly more frequent than *FLT3-ITD* mutations in inv(16) but not in t(8;21) AML patients [60].

The prognostic relevance of *FLT3* mutations in CBF-AML is controversial.

Kayser et al. reported a retrospective study of 65 CBF-AML patients with *FLT3-ITD* mutations, bearing additional cytogenetic abnormalities in 58% of cases; 24% of these patients underwent HSCT [61]. In spite of a high rate of CR (98%) after initial IC, CBF-AML patients with *FLT3-ITD* mutations had an inferior outcome as compared to previously published data on CBF-AML without *FLT3-ITD* [61]. A more recent report on this study extended to 97 *FLT3-ITD*-mutant patients better defined the prognostic impact of these mutations in CBF-AML patients. Most patients received an IC; the median follow-up was 4.43 years, and the median survival after IC and non-intensive treatment was not reached and 0.96 years, respectively [62]. Among IC-treated patients, the 4-year RFS was much better among inv(16) patients with trisomy 22 compared to the all the rest of *FLT3-ITD*-mutant patients (80% vs. 38%, respectively) [62]. Twelve patients underwent allo-HSCT in CR1 and 12 after relapse, with a survival benefit observed only for relapsed but not for patients in CR1 [62]. Thus, this study provided evidence that the outcome of CBF-AML patients with FLT3-ITD may be inferior to that of patients without *FLT3-ITD* mutations, as based on previously published data [62].

It is important to note that two recent studies based on the analysis of many cohorts of CBF-AML patients supported in most instances a negative impact of *FLT3-ITD* mutations [57,58].

In 2025, the HARMONY study proposed a new molecular classification of AML defining 17 molecular subsets, including a group of t(8;21) AML and a group of inv(16) AML patients [63]. A validation of this classification proposed additional changes, including the separation of AML patients with inv(16) plus *FLT3* mutations from the main group of AML with inv (16) [63].

### 6.3. Prognostic Impact of RAS Mutations

*RAS* mutations (mainly *NRAS*) occur frequently (30–40%) in CBF-AML, particularly in inv(16) AML, and contribute to drive increased proliferation of leukemic cells. The prognostic impact of *NRAS* mutations in CBF-AML is matter of debate in that in different studies *NRAS* mutations were found to have a variable impact on prognosis, with some studies indicating a negative effect on OS and RFS, while others showed no significant impact or benefit [58]. Studies in pediatric AML patients suggest that *NRAS* mutations in inv(16) patients were independent predictors of favorable outcomes [64]. Similarly, studies in adult CBF-AML patients treated with a FLAG (Fludarabine, Cytarabine, G-CSF) regimen showed longer RFS in *RAS*-mutant than in *RAS*-WT patients [65].

### 6.4. Prognostic Relevance of ASXL1 and ASXL2 Mutations

*ASXL1* and *ASXL2* mutations are frequently observed in t(8;21) AML patients. However, these mutations do not seem to affect outcomes of either adult [66] or pediatric [67] t(8;21) AML patients. A large survey on *ASXL2* mutations in AML patients showed that these mutations do not have a significant prognostic impact in t(8;21), while in non-t(8;21) AML the presence of *ASXL2* mutations was associated with unfavorable prognosis [68].

### 6.5. Prognostic Relevance of ZBTB7A Mutations

*ZBTB7A* mutations are observed in about 10–20% of t(8;21) AML patients, while they are undetectable in inv(16) AML patients. *ZBTB7A* mutations act as cooperating hits with the AML1-ETO fusion protein, enhancing clonal expansion by disrupting myeloid differentiation and promoting proliferation [69]. Mutations of *ZBTB7A* typically involve the C-terminal zinc-finger domain, leading to loss of DNA binding, altered cytoplasmic localization, and loss of protein anti-proliferative function [22]. Retrospective analyses on clinical studies in t(8;21) AML patients have shown that *ZBTB7A* mutations themselves have no significant impact on prognosis [70].

### 6.6. Prognostic Relevance of Cohesin Complex Mutations

Cohesin complex mutations are frequent, recurrent alterations in t(8;21) AML, affecting approximately 18–25% of these cases [19,23]. *RAD21* mutations (14%) are the most frequent, followed by *SMC3* (9.1%), *SMC1A* (8.3%) and *STAG2* (0.4%) [71]. A screening on cohesin-mutated AMLs showed that *SMC1A* mutations are enriched in t(8;21) AML compared to the rest of AML (20% vs. 3.5%, respectively) [72].

Mutations in cohesin complex genes are usually nonsense or frameshift mutations, suggesting a loss of function, reducing chromatic accessibility and gene expression, cooperating with the *RUNX1::RUNX1T1* fusion protein to inhibit cell differentiation. The prognostic impact of cohesin complex gene mutations in t(8;21) AML has been debated. Some studies suggest a general effect of cohesin complex gene mutations in increasing the CIR of t(8;21) AML [23], while other studies suggest that *SMC1A* mutations are an independent negative prognostic factor for t(8;21) AML patients [33].

## 7. Therapy-Related CBF-AML

Therapy-related CBF-AML (t-CBF-AML) is a distinct entity comprising roughly 10–15% of CBF-AML cases, often arising from treatment (DNA cytotoxic chemotherapy or radiation therapy) for prior malignancies. Patients are generally older (median age 59–54 years), more likely to be female, and have a lower white blood cell count at diagnosis compared to de novo cases [73].

Chu et al. reported a comparative analysis of the genomic profiles and outcomes in 27 t-CBF-AML patients compared to 109 CBF-AML patients [74]. The genomic profiles of t-CBF-AML and CBF-AML were highly comparable, with no significant differences in the prevalence of chromatin modifier genes, cohesin complex genes, transcription factor genes, tyrosine kinase genes and tumor suppressor genes; the frequency of myelodysplasia-related genes was higher in t-CBF-AML than in CBF-AML, but this difference was not significant [74]. Cytogenetic abnormalities and their frequencies were similar in t-CBF-AML and in CBF-AML [74].

Historically, t-CBF-AML has been associated with lower survival compared to de novo cases. However, recent studies suggest that in fit, intensively treated patients, outcomes may be more comparable when adjusted for age. Thus, Chanut and coworkers reported the results of a retrospective analysis carried out on 749 CBF-AML patients, including 78 t-CBF-AML patients, treated with IC (7 + 3 with consolidation therapy with intermediate/high-dose Cytarabine courses); 9% of patients also received GO, and 9% of t-CBF-AMF and 4% of CBF-AML patients underwent allo-HSCT [75]. No differences were observed in the CR, the relapse rate, OS and non-relapse mortality between t-CBF-Aml and CBF-AML patients [75].

Senapati and coworkers reported an exploratory analysis of two ongoing clinical trials of FLAG therapy in 219 patients with CBF-AML, of whom 32 had t-CBF-AML; the patients were treated with FLAG-IDA or FLAG-GO regimens [76]. FLAG-based therapy showed comparable results in terms of RFS and OS between t-CBF-AML and CBF-AML patients, and this was true for both FLAG-IDA and FLAG-GO treatments [76]. In t-CBF-AML patients FLAG-IDA and FLAG-GO achieved similar results in terms of RFS and OS; FLAG-GO trends toward higher rates of MRD negativity compared to FLAG-IDA [76].

## 8. Therapy of CBF-AML

The standard classical treatment of CBF-AML consisted of intensive chemotherapy typically involving 7 days of continuous intravenous Cytarabine and 3 days of anthracycline intravenous (7 + 3), followed by multiple cycles of high-dose Cytarabine consolidation [77,78]. However, this therapy has been associated with a 30–50% relapse rate [78], thus supporting the consideration of alternative induction regimens such as FLAG-IDA (Fludarabine, Cytarabine, Idarubicin, G-CSF) and induction enhancements with Gemtuzumab Ozogamicin (GO).

Following induction therapy in CBF-AML, repetitive cycles of high-dose Cytarabine are an essential component of therapy and have shown benefit in inducing prolonged remission and improving overall outcomes [2,77,78]. A randomized clinical trial showed that in AML patients with favorable prognosis, also including CBF-AML patients, consolidation therapy based on high-dose Cytarabine monotherapy was superior to standard-dose Cytarabine-based multiagent regimens [79]. Even with the development of new induction regimens, the adoption of an optimal consolidation therapy is of fundamental importance for an optimal therapeutic response in CBF-AML patients [80].

A study by Begna and coworkers provided a long-term evaluation of 70 adult CBF-AML patients (81% de novo disease and 19% therapy-related) treated with 7 + 3 chemotherapy; 98.5% of these patients achieved remission, with 74.3% CR and 24.3% CRi. Among patients with CR/CRi, 10 patients underwent allo-HSCT in CR1 and 14 patients in CR2 [81]. After a median follow-up of 46 months, 45% relapses and 44% deaths were documented; the 1-, 3-, 5- and 10-year OS rates were 90%, 70%, 61% and 50%, respectively [81].

CBF-AML comprises about 5% of AML cases diagnosed in individuals over 60 years old. Intensive chemotherapy treatment can be administered to a part of older CBF-AML patients fit for intensive treatments. Thus, a recent study by Mosna et al. reported the outcomes of 219 older (>60 years) CBF-AML patients receiving an IC induction treatment: 88% of these patients received an anthracycline during induction [82]. A CR was achieved in 84% of cases (86% vs. 78% for younger (<70 years) and older (>70 years) patients, respectively); 5-year OS was 44% (48% for patients <70 years and 33% for those >70 years) [82]. A part of patients who achieved CR also received consolidation treatment with cytosine arabinoside [82]. Although more fit elderly CBF-AML patients may benefit from conventional IC induction, it is evident that many older CBF-AML patients are not eligible for intensive treatments due to age-related frailties and require to be treated using less intensive treatment approaches.

Other studies have evaluated alternative induction regimens. An initial single-institution retrospective study showed that, in a group of 114 CBF-AML patients, the treatment with FLAG or FLAG-IDA improved EFS compared to standard 7 + 3 chemotherapy [83]. In a more recent study, in a group of 109 ND adult CBF-AML patients, a 5-year RFS of 59% and a 5-year OS rate of 68% were reported [84]. In the FLAG-IDA regimen used in this study, a total of 12 mg/m^2^ of Idarubicin was used, a dose lower than that used in other studies, to lower the myelotoxicity of this regimen [84]. Interestingly, using the FLAG-IDA regimen, the negative impact of mutations of *KIT*, *FLT3* and *RAS* was not observed [83]. In this study 65 additional CBF-AML patients received the FLAG regimen in association with GO; the FLAG-GO association resulted in a better RFS compared to FLAG-IDA (78% vs. 59%, respectively) [84].

A recent retrospective multicenter study on 875 pediatric AML patients compared the efficacy of FLAG-IDA to DAE (Daunorubicin, Cytarabine, Etoposide) [85]. In the whole cohort of patients FLAG-IDA improved OS compared to DAE (5-year OS 92.4% vs. 88.9%, respectively); a similar observation was made in CBF-AML patients (5-year OS of 90.4% for FLAG-IDA and 78.4% for DAE) [85].

### 8.1. Gemtuzumab Ozogamicin (GO) for the Treatment of CBF-AML

Gemtuzumab Ozogamicin (GO) is an antibody–drug conjugate targeting the membrane antigen CD33, markedly expressed in the majority of AML cases. This drug is composed of a monoclonal antibody anti-CD33 conjugated with the cytotoxic agent calicheamicin; following its binding on the surface of leukemic blasts, the complex between GO and CD33 is internalized and then released into the cells, where calicheamicin exerts its toxic effects, inducing cell death. GO was first approved for AML treatment in 2000. However, concerns about the induction of increased mortality led to its market withdrawal in 2010 [86,87]. The AML 15 trial enrolled 137 CBF-AML patients, 72 of whom received GO and provided evidence that the addition of GO to induction chemotherapy induced an improvement in OS, particularly evident in favorable-risk AML [88]. Following the introduction of dose fractionation, the ALFA-0701 trial showed an improvement of EFS, RFS and OS in 272 treatment-naïve AML patients receiving GO in addition to IC [89].

A meta-analysis based on the evaluation of five randomized clinical trials, involving a total of 3325 AML patients, showed a benefit deriving from the addition of GO, pronounced for patients with genetically favorable AML; particularly, for CBF-AML patients, a 22.5% of absolute OS benefit at 5 years compared to IC alone was observed [90].

On the basis of the results observed in these studies, GO was reapproved by the FDA in 2017 and the EMA in 2018 for first-line treatment of CD33-expressing AML [87].

A recent retrospective analysis confirmed these observations. Thus, Ronnacker and coworkers retrospectively analyzed 265 CBF-AML patients treated in the context of the SAL, AML CG and CELL cooperative study groups, showing that patients treated with 7 + 3 induction chemotherapy plus GO had a 2-year OS of 90% compared to 80% in those without GO [91]. The 2-year EFS was 51% in the GO group and 36% in the IC-alone group [91]. Complete remission rates were comparable in the group of patients (89% with GO and 90% with IC alone) and the frequency of MRD negativity was significantly higher in GO than in the IC-alone group (77% vs. 49%, respectively), resulting in a reduced cumulative incidence of relapse [91] (Table 2). The survival benefit was equally observed for *CBFB::MYH11* and *RUNX1::RUNX1T1* patients. Importantly, survival outcomes were similar regardless of whether GO patients received one or two versus three doses of GO [91]. The median time to MRD negativity was significantly shorter in GO vs. non-GO patients at 5.6 and 8.4 months, respectively [91]. Finally, high-grade toxicities were not increased in the group of GO-treated patients compared to IC alone [91]. In conclusion, this study showed that the OS benefit deriving from the addition of GO to IC appeared primarily attributable to a reduced risk of relapse rather than higher remission rates. These observations support the integration of GO into treatment algorithms for patients with CBF-AML.

The effectiveness of GO in the treatment of CBF-AML has also been supported by clinical studies performed using the FLAG regimen (Fludarabine, Cytarabine, G-CSF); in one study the FLAG-GO regimen was compared to the standard FLAG-IDA (Idarubicin) regimen. A first study in 2014 reported a high rate of remission (95%), with 5% induction deaths in 45 CBF-AML patients with a median age of 48 years; OS and RFS at 3 years were 78% and 85%, respectively [93]. A more recent study reported the results on 174 CBF-AML patients in the context of a prospective clinical trial of FLAG-based induction/consolidation in combination with GO (FLAG-GO, 65 patients) or in combination with Idarubicin (FLAG-IDA, 109 patients) [83]. The 5-year RFS was significantly better in FLAG-GO compared to FLAG-IDA (78% vs. 59%, respectively); there was a strong trend for better OS with FLAG-GO compared to FLAG-IDA (p 0.06) [94]. The FLAG-GO regimen was superior to the FLAG-IDA regimen in inducing optimal disease-specific transcript reduction at different times during treatment. In a multivariate analysis for RFS, several significant variables were observed, including lower age, 4-log reduction of CBF-related fusion transcript by qRT-PCR at the end of consolidation therapy and additional cytogenetic abnormalities [83]. A more recent analysis of this study extended to 200 CBF-AML patients (92 treated with FLAG-GO and 108 with FLAG-IDA) provided evidence that FLAG-GO significantly improved the rate of patients achieving optimal PCR response to therapy in terms of the decrease of fusion transcript levels (at the end of treatment, 92% in FLAG-GO and 61% with FLAG-IDA); FLAG-GO significantly improved OS compared to FLAG-IDA (5-year OS 79% and 69%, respectively); and FLAG-GO significantly improved RFS compared to FLAG-IDA (5-year RFS 77% vs. 59%, respectively) [94].

A phase II clinical trial (NCT 008014989) evaluated 219 frontline patients with CBF-AML: 51% received FLAG-GO and 49% FLAG-IDA. Patients were evaluated for response and for dynamics of MRD, assessed at diagnosis, at the end of IC and during and after consolidation therapy (optimal response (OPR) was defined as ≤0.1% positivity after IC and ≤0.01% after consolidation therapy) [92]. Baseline mutations of these patients included 49% TRK pathway, 44% MAP Kinase, 11% *DNMT3A-ASXL1-TET2* and 7% transcription factors [92]. After IC, 47.7% of patients achieved OPR, including 61.6% of patients treated with FLAG-GO and 34.1% of patients treated with FLAG-IDA; after consolidation therapy, 53% of patients achieved OPR, including 72.8% of patients treated with FLAG-GO and 29.4% of patients treated with FLAG-IDA [92]. The achievement of OPR predicted a better RFS but comparable OS with respect to those without an OPR. Among patients who achieved early OPR, the 5-year OS was better than in those achieving a late OPR (91.3% vs. 70.4%, respectively); a higher proportion of patients treated with FLAG-GO achieved an early OPR compared to those treated with FLAG-IDA (61% vs. 34%, respectively) [92]. The 5-year RFS rate was 75.2% vs. 60.6% in patients with inv(16) treated with FLAG-GO and FLG-IDA, respectively, while the 5-year OS was 81.5% vs. 76% [92]. Among patients with t(8;21) the 5-year RFS rate was 78.1% vs. 58.1%, while 5-year OS was 78.7% vs. 61.6% among FLAG-GO- and FLAG-IDA-treated patients, respectively [92] (Table 2). Thus, treatment with FLAG-GO was associated with early and higher rates of deepening responses with time [92]. Factors predicting a better OPR after IC and consolidation therapy were FLAG-GO and inv(16), while the mutational profile did not affect the achievement of OPR [92]. The 5-year RFS rates were 79.5% vs. 59.4% in the FLAG-GO and FLAG-IDA-treated patients, respectively; the 5-year OS rates were 80.1% and 69.5%, respectively, in the FLAG-GO and FLAG-IDA-treated patients; and the 5-year CIR was 17.2% with FLAG-GO and 28.3% with FLAG-IDA [92]. Among patients ≥60 years, CIR rates were lower with FLAG-GO compared to FLAG-IDA (15.1% vs. 37.5%, respectively) [92] (Table 2). Among FLAG-GO-treated patients, the 5-year OS rate was 85% in patients <60 years and 71.6% in patients ≥60 years, while it was 75.5% and 48.6% in patients treated with FLAG-IDA, respectively [92]. The mutational profile did not affect the response to FLAG-IDA and FLAG-GO; particularly, both *KIT*-mutated and *KIT*-WT patients displayed a comparable response to FLAG-IDA and FLAG-GO [92]. Patients with additional chromosomic abnormalities had a lower RFS but not OS compared to patients without additional chromosome abnormalities [92]. Multivariate analysis of the variables affecting the response of patients showed that for both RFS and OS only treatment with FLAG-GO and at age <60 years was favorable, while CBF-AML type and mutational groups were not significant [92]. In conclusion, the results of this study showed that: survival outcomes were significantly superior in patients who received FLAG-GO compared to FLAG-IDA; baseline myeloid mutations did not impact outcomes, including achievement of OPR; and even in patients achieving an OPR, FLAG-GO-treated patients displayed better survival outcomes than FLAG-IDA-treated patients.

A HARMONY database from Europe included 520 annotated AML cases with t(8;21) treated with 7 + 3 alone (87% of cases) and 7 + 3 chemotherapy + GO (13% of cases) [48]. A multivariate analysis showed that the addition of GO to IC was a factor significantly improving prognosis [48].

### 8.2. Venetoclax and Hypomethylating Agents for the Treatment of CBF-AML

Venetoclax (VEN)-based regimens have been evaluated in CBF-AML patients either as frontline therapy or in relapsed/refractory (R/R) patients or as post-transplant therapy.

Some studies evaluated VEN as first-line therapy (Table 3).

Only two studies reported the association of VEN with chemotherapy. Thus, Wang et al. explored VEN plus modified-intensity Idarubicin and Cytarabine treatment as first-line therapy for newly diagnosed pediatric AML patients [95]. A total of 65 patients were enrolled in this study. After cycle 1, CRR and MRD negativity were 90.8% and 78.5%, increasing to 96.8% and 87.3% after induction cycle 2; 28 patients underwent HSCT. Patients with CBF-AML (t(8;21) and inv(16) achieved a favorable response rate [95]. However, molecular monitoring of the response showed a median log10 reduction of leukemic-specific transcripts suboptimal (−1.7 after cycle 1 and 2.6 after cycle 2 for t(8;21) and −2.3 and 2.5 for inv(16)) [92]. In line with this last observation, disease relapse was frequently observed in t(8;21) and inv(16) patients with *KIT* mutations [95]. In the whole population of treated leukemic patients, 12-month OS and EFS were 92.3% and 79.1%, respectively [95].

A recent study reported a retrospective analysis on 60 newly diagnosed CBF-AML adult patients treated with VEN combined with IC as induction therapy [96]. The ORR with CR rate after one cycle of treatment was 93.3%; no differences were observed between t(8;21) and inv(16) patients. The 2-year OS, EFS and RFS rates were 86.6%, 80% and 90%, respectively [96]. No patient death occurred during treatment.

A Myelo-MATCH treatment trial (NCT06917911) will compare the effect of Ven to GO when given with the 7 + 3 chemotherapy regimen for the treatment of patients with CBF-AML.

Several studies have evaluated VEN+HMA in AML patients with t(8;21) or inv(16) AML patients (Table 3). Patients with CBF-AML were excluded from the VIALE trial on VEN+HMA in elderly AML patients. An initial study by Arslan and coworkers explored the safety and the efficacy of VEN+HMA in a group of 46 AML patients with a mean age of 70 years with a favorable risk profile, including 10 CBF-AML patients; among CBF-AML patients, a CR+CRi rate of 80%, comparable to the response rate observed in the rest of favorable risk patients, was observed [97].

**Table 3:** Main clinical studies based on the use of VEN in CBF patients.

Reference	Number of Patients Age	Type of Patients	Type of Treatment	Complete Remission	Overall Survival
Wang et al., 2025 [95]	65 Pediatric patients	t(8;21) inv(16)	VIA regimen VEN+modified-intensity Idarubicin and Cytarabine	92.5%	96.8%
Ruhnke et al., 2025, 2026 [98,99]	66 Median age 76 yrs	t(8;21) 34 pts inv(16) 32 pts	VEN+ Azacitidine	69%	14 mo
				t(8;21) 68%	t(8;21) 13 mo
				inv(16) 71%	inv(16) 23 mo
Shen et al., 2025 [100]	142 36–42 years	t(8;21) 85 pts inv(16) 57 pts	VEN+HMA 72 pts IC 70 pts	inv(16)	inv(16)
				VEN 94.9%	VEN 8.7 mo
				IC 88.9%	IC 5.6 mo
				t(8;21)	t(8;21)
				VEN 51.5%	VEN 7.1 mo
				IC 82%	IC 9.1 mo
Feng et al., 2025 [101]	44 Mean age 49 yrs ND 51 yrs R/R	37 ND 7 R/R Inv(16)	VEN+Azacitidine 65% VEN+Decitabine 35%	ND 2yrs	ND 100% R/R 57%

Zhang et al. reported the retrospective analysis of 30 CBF-AML patients (13 with AML1-ETO and 17 with *CBFB::MYH11*), with a mean age of 40 years, treated with VEN+HMA regimens [102]. Among patients with AML1-ETO fusion, 5/13 achieved a CR+CRi; among patients with *CBFB::MYH11* fusion, all patients (17/17) achieved a CR+CRi [102].

Ruhnke and coworkers reported the results of a real-world retrospective study involving 53 patients with ND CBF-AML (27 with inv(16) and 26 with t(8;21) AML), with a median age of 76 years, treated with VEN+HMA [98]; 20% of these patients had post-cytotoxic t-CBF-AML. Recently, this study was updated to 66 patients (32 t(8;21) and 34 inv(16)) [99]. *NRAS*, *TET2*, *KIT*, *NRAS* and *DNMT3A* were the most common co-mutations observed in these patients and were comparable in the two groups of patients, except for *KRAS* mutations more frequent in patients with inv(16) than with t(8;21) (44% vs. 5%, respectively) [98]. The CRc rate for the whole cohort was 69% and similar for Inv(16) or t(8;21) fusions [68% vs. 71%, respectively); the mOS for the entire cohort was 14 months, with 12-month and 24-month OS rates of 55% and 40%, and 13 months for t(8;21) patients and 23 months for inv(16) patients [98,99]. The presence of *NRAS*, *KRAS*, *FLT3-ITD* and *KIT* mutations did not adversely affect mOS [98,99]. Two patients underwent allo-HSCT. With a mOS of 14 months, outcomes appeared to be less favorable than those previously reported for patients with ELN2024 favorable-risk disease and similar to those observed in ELN2024 intermediate-risk non-CBF AML patients; according to these observations, it was proposed that patients with CBF-AML treated with VEN-based regimens should be classified as having intermediate-risk disease [99].

Shen and coworkers reported a retrospective analysis on 142 CBF-AML patients, 70 treated with an Idarubicin-based regimen (IC) and 72 treated with VEN-based regimens, with a median age between 36 and 42 years [102]. In the inv(16) cohort, 39 patients received VEN regimens and 18 IC; in the t(8;21) cohort, 33 patients received VEN regimens and 52 IC [100]. Among inv(16) patients, in the VEN group CR rates were 94.9% and 88.9% in the IC group; among t(8;21) patients, in the VEN group 51.5% of patients achieved a CR, and 82% were in the IC group [98]. In patients with *KIT*, *FLT3-ITD* or *NRAS* mutations, a CR rate was achieved in 96.6% of patients of the VEN group and 100% of the IC group; in those without *KIT*, *FLT-ITD* or *NRAS* mutations the CR rate was 90% in the VEN group and 77.8% in the IC group [100]. In the group of t(8;21) but not of inv(16) patients, the presence of *KIT*, *FLT3-ITD* or *NRAS* mutations was associated with a decreased rate of CR following VEN treatment. In the inv(16) patients who underwent HSCT, the mOS was 16.4 months in the VEN and 25.8 months in the IC group; among patients who did not undergo HSCT, the mOS was 8.7 months in the VEN and 5.6 months in the IC group, respectively [98]. In t(8;21) patients who underwent HSCT, the mOS was 20.3 months and 20.1 months in the VEN and IC groups, respectively; among patients who did not undergo HSCT, the mOS was 7.1 months vs. 9.1 months in the VEN and IC groups, respectively [100]. In conclusion, this study showed the same efficacy and greater safety of VEN compared to IC in adult AML patients with CBF-AML, except for t(8;21) AML patients with kinase mutations.

Feng and coworkers reported a study of 37 newly diagnosed and seven relapsed inv(16) AML patients treated with VEN+HMA (65% VEN+Azacitidine and 35% VEN+Decitabine); the mean age of newly diagnosed patients was 49 years and of relapsed patients was 51 years [101]. A total of 100% of newly diagnosed patients and 57% of relapsed patients achieved a CR+CRi; 2-year OS was 97.2% and 2-year RFS 71.3% for newly diagnosed patients. Five of these patients experienced relapse after a median of 11 months, and 12 proceeded to allo-HSCT [101]. In almost all newly diagnosed patients, inv(16) transcripts markedly declined after the first cycle of VEN therapy, and after the second cycle of treatment, the mean transcript of fusion gene was 18 copies/cell, with a minority of patients displaying undetectable fusion transcript levels [101].

Wu et al. reported the retrospective analysis of 203 patients diagnosed with CBF-AML (115 with t(;21) and 88 with inv(16) at a West China Hospital, treated either with standard intensive chemotherapy (IC 123 patients) or VEN-based therapy (VEN 80 patients); patients treated with VEN-based regimens received either VEN+HMA or VEN+chemotherapy [103]. Among the t(8;21) patients, VEN resulted in a CR rate of 75% compared to 66% in IC patients; among inv(16) patients, CRc rates were 89.1% with VEN vs. 84.1% with iC, respectively [103]. mOS was similar between VEN and IC patients and for VEN patients was similar for t(8;21) and inv(16) [103].

In a retrospective analysis, Shu and coworkers evaluated 16 *KIT*-mutant patients receiving VEN+HMA, 141 *KIT*-WT patients receiving VEN+HMA and 69 *KIT*-mutant patients receiving IC [104]. Patients with *KIT* mutations displayed a poor response and shorter EFS to VEN+HMA compared to either *KIT*-WT treated with VEN+HMA or *KIT*-mutant treated with IC [104].

Shi et al. recently reported a retrospective analysis of 26 inv(16) AML patients with molecular relapse or hematological relapse who received a treatment based on a VAG regimen: VEN at 400 mg/day from day 1 to 21, Azacitidine at 75 mg/m^2^/day from day 1 to day 7 and G-CSF at 5 μg/kg/day from days 1 to 7 [105]. After three cycles of VAG treatment, 18/26 (69.2%) patients achieved a molecular response, including 50% who attained MRD negativity and 19.2% who achieved a greater than 0.5-log reduction in *CBFB::MYH11* transcript levels [105]. After a median follow-up of 7.2 months, no death occurred with an estimated 2-year OS of 100% and a 2-year EFS of 71.1% [105].

## 9. Evaluation of Minimal Residual Disease in CBF-AML Patients

Measurable residual disease (MRD) monitoring has become a critical component in the management of AML to inform prognosis, guide therapy, and serve as a key end point in clinical trials. Evaluation of MRD using multiparametric flow cytometry (MFC), real-time quantitative polymerase chain reaction (RT-qPCR), or next-generation sequencing represents a fundamental tool in the management on intensively treated AML patients.

MRD may be detected using different techniques. One approach is based on a multiparametric flow cytometry technique for rapid information about the residual leukemic burden during or after the end of induction therapy; however, the main limitations of this technique are related to the lack of specificity and to the limited sensitivity (10^−3^–10^−4^) to detect minimal numbers of residual cells [106]. Molecular techniques, such as quantitative polymerase chain reaction (qPCR) or digital PCR, are much more sensitive (10^−6^) and specific but are only suitable for patients with specific molecular aberrations, such as CBF translocations [106]. NGS had a sensitivity comparable to PCR and offers the advantage of the possible detection and monitoring of multiple genetic alterations [106]. The ideal technique for MRD monitoring of AML patients in a clinical context is provided by a specific and sensitive technique of MRD detection [106].

Regardless of the technique of evaluation of MRD, AML patients who are in morphological CR with MRD positivity have a higher risk of relapse and poor prognosis compared to those who are in CR with MRD negativity. Many AML patients, including those with CBF-AML, have a molecular marker of leukemic disease and offer the opportunity for molecular monitoring of their disease status with high specificity and sensitivity during treatment.

Long-term molecular monitoring of MRD in CBF-AML patients was shown to be predictive of relapse. Thus, in the CBF-2006 study, a prospective monitoring of peripheral blood and bone marrow samples was performed every 3 months and every year, respectively, for 2 years following IC in 94 patients with t(8;21) AML patients [107]. After competition of consolidation therapy, a bone marrow complete remission (defined as a *RUNX1::RUNX1T1* positivity of <0.001%) was observed in 30% of patients but was not predictive of subsequent relapse [107]. Conversely, a peripheral blood complete molecular remission was associated with a lower risk of relapse whatever the time-point considered after consolidation therapy [107]. During a 2-year follow-up, the persistence of PB MRD negativity was associated with a 4-year cumulative incidence of relapse of 8.2%, while MRD-positivity was associated with a 4-year cumulative incidence of relapse of 86.9% [107]. The AML Study Group confirmed the findings of this study, showing in a group of 155 intensively treated t(8;21) AML patients that achievement of >2.5-log decrease after cycle 1 treatments and >3.0-log decrease after cycle 2 were significantly associated with a reduced risk of relapse; after competition of therapy, MRD negativity in both BM and PB was associated with a significant improvement of mOS (4-year OS 93% compared to 70% in patients not achieving MRD negativity) [108].

Clinically validated assays for the detection of molecular MRD are available for MRD evaluation in t(8;21) or inv(16) patients, and their use is highly recommended [109].

There is a strong rationale to try to eradicate residual disease remaining in CBF-AML patients after IC or HSCT using low-intensity treatments based on agents such as VEN or hypomethylating drugs. In this context, Tiong et al. reported the results of a prospective phase II study evaluating the safety and the efficacy of VEN combined with low-dose Cytarabine (VALDAC) in AML patients at first MRD or oligoblastic relapse [110]. The study enrolled 48 patients, including one patient with t(8;21) and one patient with inv(16) [110]. In the whole population of AML patients VALDAC treatment elicited a log_10_ reduction, with 46% of MRD negativity in the MDR-relapse group; the 2-year OS rate was 67% in the MDR group and 53% in the oligoblastic group [110]. Concerning the two CBF-AML patients enrolled in this study, the inv(16) patient achieved an MRD negativity, while the t(8;21) oligoblastic patient was resistant to the VALDAC treatment [110].

Bazinet reported the results of a single-center, single-arm phase II study enrolling 35 AML patients in CRc following IC (25 patients) or low-intensity induction and not immediately eligible for HSCT [111]. The study enrolled a total of six CBF-AML patients, four with inv(16) and two with t(8;21) AML [111]. These patients were treated with VEN+AZA and received a median number of nine cycles of treatment [111]. In the whole population of AML patients, the 2-year RFS was 65%, 71% in cohort 1 and 52% in cohort 2 [111]. Concerning the six CBF-AML patients, three of six experienced AML relapse (two patients with inv(16) and one patient with t(8;21)); these relapsing patients were heavily pretreated high-risk patients, with two being MRD-positive at enrollment [111].

Medawar et al. confirmed the efficacy of VEN+HMA in a group of 25 AML patients who achieved a CRc condition after IC induction; two of these patients had CBF-AML. Many patients proceeded to HSCT and had an RFS not reached compared to 11.2 months for those who did not proceed to HSCT [112].

Orvain and coworkers explored a group of 303 patients with *NPM1-m* AML or CBF-AML (t(8;21) 199%, inv(16) 27% and *NPM1-m* 53%) who were in first CR and who had MRD monitoring after first-line IC [113]. Of all the patients, 18% of t(8;21) and 16% of inv(16) AML cases relapsed; the cumulative incidence of relapse was lower in t(8;21) AML than in inv(16) (23% vs. 31%, respectively). Furthermore, molecular relapse occurred later in t(8;21) patients than in inv(16) patients (median 377 days vs. 269 days, respectively) [108]. Importantly, patients who received preemptive therapy at times of molecular MRD relapse had better OS than those treated for morphological relapse [113]. In another study, the same authors reported the evaluation of 70 patients (mostly *NPM1-m* and only four with CBF-AML) with molecular MRD relapse treated with VEN+AZA, and all patients achieved either a CR^MRD−^ or a CR^MRD-LL^ (with marked lowering of MRD positivity) [114].

Other studies have explored the capacity of hypomethylating agents, such as Azacitidine (AZA) or Decitabine (DEC), as maintenance therapy in CBF-AML patients. In a first study, Ragon and coworkers evaluated AZA and DEC in 23 CBF-AML patients who received HMA therapy following induction/consolidation therapy with FLAG-IDA+GO; of these 23 patients, 17 patients had detectable MRD at HMA initiation [115]. Of these 23 patients, five had progressive disease, seven did not respond to HMA and 11 with a median MRD positivity at an HMA initiation of 0.06 displayed a significant decrease in residual disease, particularly evident during cycle 1 and 2 of treatment with HMA [115].

In a second study, 31 CBF-AML patients received DEC as maintenance therapy after FLAG-IDA induction/consolidation treatment; 48.3 of these patients completed FLAG treatment but had an MRD positivity at the end of treatment (Group 1). A total of 16 patients could not complete the consolidation treatment and were switched to DEC maintenance: eight of these patients had undetectable MRD (group 2A), and the other eight patients had detectable MRD (Group 2B) [116]. In group 1 of patients, 10/15 patients cleared the fusion transcript during DAC maintenance and remained in complete molecular response (CMR) during maintenance, while the remaining five patients did not attain MRD negativity and one of these patients relapsed; six of these patients needed salvage therapy based on chemotherapy alone or chemotherapy + HSCT or HSCT. The median estimated OS of these patients was 114 months [116]. In group 2A of patients, 6/8 patients remained in remission during DEC maintenance therapy, and 2/8 relapsed during DEC maintenance therapy and need salvage therapy; these patients had an estimated OS of 101.9 months [116]. In group 2B of patients, 3/8 displayed a marked reduction in MRD positivity, but all of these had molecular relapse; all the patients of this group needed salvage therapy and five proceeded to HSCT. The median estimated OS was 34 months [116]. Amongst the 23 patients who had a PCR ≥0.01% before maintenance therapy, 12 patients attained a CMR: the pre-DEC values of PCR were 0.003% in patients with CMR compared to 0.14% in non-responders [116]. These studies support the potential benefit deriving from maintenance therapy with HMA in CBF-AML patients who have been treated with complete cycles of induction/consolidation chemotherapy and with low residual disease post-therapy.

## 10. Hematopoietic Stem Cell Transplantation for CBF-AML

The 2022 European Leukemia Net (ELN) guidelines recommend reserving allo-HSCT for patients with relapsed or refractory CBF-AML [117]. However, for these patients, allo-HSCT remains the best chance for long-term leukemia-free survival.

For patients with relapsed CBF-AML the prognosis is generally favorable compared to other AML subtypes, but survival highly depends on achieving a second remission and undergoing transplantation. Using salvage chemotherapy (such as high-dose cytarabine regimens) up to 70% of relapsed CBF-AML patients can achieve a second complete remission (CR2), but their long-term survival without subsequent HSCT is significantly lower: for patients treated with chemotherapy alone the long-term survival was estimated to be <30%, compared to a long-term survival of 45–60% for patients achieving a CR2 with salvage chemotherapy and then receiving allo-HSCT [118,119,120].

Konuma and coworkers retrospectively analyzed 959 adult patients with t(8;21) or inv(16) AML treated by allo-HSCT in complete remission: 49.3% of t(8;21) AML patients were transplanted at CR1 and 50.7% at CR2, and 31.4% of inv(16) patients were transplanted at CR1 and 68.6% at CR2 [115]. In t(8;21) patients, the probability of OS at 4 years was 62.1% for MDR^pos^ patients and 69.5% for MDR^neg^ patients, while in inv(16) patients the 4-year OS was 74.6% for MDR^pos^ and 74% for MRD^neg^ patients {116]. In the multivariate analysis, in t(8;21) AML patients only MDR^neg^ status was associated with mortality and MDR^pos^ status, and CR2 at HSCT was significantly associated with treatment failure; in the multivariate analysis, in inv(16) AML patients MRD status was not associated with overall mortality or treatment failure [121]. Among patients undergoing HSCT during CR1, MRD status did not affect relapse or survival in either t(8;21) or inv(16) AML in either t(8;21) and inv(16); in contrast, among t(8;21) patients undergoing HSCT during CR2, MDR^pos^ status was significantly associated with poorer OS and RFS and a higher relapse rate [121]. Multivariate analysis confirmed that MRD^neg^ status was associated with lower treatment failure and relapse in t(8;21) AML patients [121]. Thus, this study showed that the pretransplant MDR positivity was significantly associated with a higher incidence of relapse and inferior leukemia-free survival only in patients with t(8;21) undergoing HSCT during CR2. This finding strongly supports the need to develop efficient strategies for t(8;21) patients with MRD^pos^ at CR2, including chemotherapy prior to HSCT, the optimal timing of HSCT and the use of post-transplant maintenance therapy to prevent relapse.

Recently, Huang and coworkers confirmed and extended these findings in a study reporting the analysis of 825 de novo CBF-AML Chinese patients who received treatment with standard induction and consolidation chemotherapy [122]. Three-year OS was 80.6% in the t(8;21) group and 90.2% in the inv(16) group. At consolidation 2 (PC2), 42.5% of t(8;21) and 33.2% of inv(16) patients were responders (MDR^neg^); 26.6% t(8;21) patients and 18.6% inv(16) patients were MDR^pos^ at PC2. In patients treated with chemotherapy alone the relapse rate was similar among t(8;21) and inv(16) patients (20.3% and 21.4%, respectively); relapse rates after HSCT were 7.2% for t(8;21) patients and 4.9% for inv(16) patients [122]. The analysis of MRD at PC2 showed that its status predicts survival in t(8;21) but not in inv(16) AML patients; among t(8;21) patients, an MDR^neg^ at PC2 showed a significantly better 3-year OS than an MDR^pos^ (86.3% vs. 76.5%, respectively). However, in inv(16) the 3-year OS was similar in those with MDR^neg^ or with MDR^pos^ at PC2 (95.1% vs. 89.9%, respectively) [122]. The analysis of the contribution of allo-HSCT to the survival of CBF patients showed that it improved outcomes in t(8;21) patients but not in inv(16) patients: in the whole population of CBF-AML patients who underwent allo-HSCT, allo-HSCT significantly improved 3-year OS in t(8;21) but not in inv(16) patients. In t(8;21) patients with an MDR^neg^ status at PC2, survival outcomes showed a trend of improvement with allo-HSCT compared to the chemotherapy group (87.5% vs. 77.3%, respectively). In inv(16) patients with either MDR^neg^ or MDR^pos^ status at PC2, allo-HSCT did not improve OS compared to chemotherapy [122,123]. A last set of analyses explored the effect of HSCT timing on outcomes; for this analysis, the patients were stratified into three subgroups, showing that among t(8;21) patients, those receiving allo-HSCT at CR1 showed significantly better 3-year OS than those receiving allo-HSCT at CR2 or with no remission and in those receiving chemotherapy alone (85.8% vs. 71.4% vs. 64.9%, respectively). In contrast, 3-year OS did not significantly change among the three subgroups of inv(16) AML patients [122,123]. Finally, a multivariate analysis showed that the presence of some clinical or biological parameters (age and WBC counts at diagnosis and presence of *KIT^816^* or *KIT^822^* mutations) were independent predictors of MDR^pos^ at PC2; based on these parameters and other factors, a risk scoring system was developed allowing the identification of a subset of high-risk t(8;21) patients who may benefit from early consideration of allo-HSCT [122,123]. These findings indicated that for inv(16) AML patients, MRD evaluation post-consolidation 2 does not provide clinically relevant information, with allo-HSCT reserved for CR2 in these patients; in contrast, t(8;21) AML patients with post-C2 MDR positivity should receive allo-HSCT in CR1 [122,123].

Allo-HSCT is recommended for CBF-AML patients who have reached a CR2. However, a recent study by Tarantino and coworkers retrospectively evaluated the outcomes in 610 patients with CBF-AML (124 inv(16) and 486 t(8;21)) who underwent allo-HSCT in R/R with active disease [124]. Graft sources included matched sibling, matched unrelated donors and haploidentical donors. Two-year OS and LFS were 53.6% and 42.7%, respectively; patients with t(8;21) exhibited higher relapse incidence and poorer survival than those with inv(16) [124]. These observations support the therapeutic role for allo-HSCT in R/R CBF-AML patients in non-CR.

Data from the Center for International Blood and Bone Marrow Transplant Research (CIBMTR) show that patients transplanted with AML not in CR, either with matched related donors or matched unrelated donors, have worse survival compared with the transplants in CR1 or CR2 cohorts [125]. These findings support that it is better to be transplanted in CR1 or CR2 compared with relapsed disease [125].

Although transplanting in CR1 or CR2 is ideal for patients with relapsed CBF-AML, advancing directly to allo-HSCT may represent an alternative without waiting for a second complete remission, which can still offer the opportunity for long-term survival for patients with active chemoresistant disease.

Post-transplant MRD monitoring in CBL-AML patients is essential, with its positivity predicting significantly worse overall survival and higher relapse risk. The current guidelines strongly recommend MRD evaluation pre- and post-transplant, particularly in AML patients with molecularly evaluable MRD, including CBF-AML [126]. Post-HSCT evaluation in CBF-AML patients requires serial assessments of *RUNX1::RUNX1T1* or *CBFB::MYH11* transcripts using qRT-PCR at various times after transplantation (particularly around neutrophil engraftment (day 30–50) and continuing for up two years) [127].

Tokuda et al. evaluated the prognostic impact of pre- and post-transplant MRD evaluation on transplant outcomes in a group of 96 CBF-AML patients who underwent allo-HSCT [128]. According to the evaluation of fusion transcript levels by qRT-PCR, they defined three different levels: MDR^neg^, MDR^wp^ (weakly positive, 30–200 copies/μg RNA) and MDR^sp^ (strongly positive, >200 copies/μg RNA) [122]. The two-year OS post-HSCT of patients with MDR^wp^ was similar to that of MDR^neg^ patients (81.5% vs. 84.5%, respectively), while those with MDR^sp^ have a worse OS and higher relapse risk [128].

The presence of MRD positivity after HSCT in CBF-AML suggests the need for a possible maintenance therapy aiming to clear or to lower residual disease post-transplantation. As discussed above, treatments with hypomethylating agents have shown some efficacy in CBF-AML patients with MDR positivity post-induction/consolidation therapy [129,130]. Two recent studies reported ab analysis of the safety and the efficacy of hypomethylating agents as post-HSCT maintenance therapy in CBF-AML patients [129,130]. Guo and coworkers performed a prospective study comparing the safety and the effectiveness of epigenetic agents (Azacitidine or Chidamide, a selective histone deacetylase inhibitor) used as prophylactic therapy in 30 high-risk t(8;21) AML patients and as preemptive therapy in 34 high-risk t(8;21) patients [129]. Prophylactic treatment with Azacitidine or Azacitidine was administered to patients with negative MRD status post-allo-HSCT; preemptive therapy was initiated for patients experiencing early molecular relapse without hematological relapse [129]. The two-year RFS and OS were significantly higher in the prophylactic group compared to the preemptive group (82% vs. 51.3%; 86.4% vs. 56.1%) [129]. Lu and coworkers reported the efficacy of Azacitidine maintenance preemptive therapy to clear MRD in five of seven CBF-AML patients with evidence of early molecular relapse post-HSCT [130].

## 11. Conclusions

The studies carried out in the last two decades have strongly contributed to better define the molecular heterogeneity of CBF-AML, with the identification of t(8;21) and inv(16) as two genotypically distinct AML subtypes. Furthermore, the characterization of t(8;21) and inv(16) AMLs allowed the identification of numerous gene mutations and chromosome abnormalities; particularly in t(8;21) AML, some of these genetic abnormalities negatively impact on outcomes.

Although CBF-AMLs are considered leukemias associated with a favorable risk profile and are highly responsive to standard induction chemotherapy, a high proportion of these patients relapse. Thus, a first important strategy to improve the outcomes of these patients consisted of improving the effectiveness of induction regimens. In this context, clinical studies based on the association of GO with standard 7 + 3 induction or a FLAG regimen showed higher rates of molecular responses with MRD negativity and a reduction in relapsing patients. Thus, 7 + 3 + GO or FLAG-GO represent the current standard of care for fit CBF-AML patients.

In spite of this progress, part of CBF-AML patients relapse and need salvage treatment. The current view is to induce a second remission in these relapsing patients and to proceed to allo-HSCT at CR2. However, recent evidence supports anticipating allo-HSCT at CR1 in subgroups of CBF-AML patients, particularly some t(8;21) AML patients considered at high-risk.

Several relevant clinical questions will need to be addressed in future clinical studies:

- Long-term evaluation of CBF-AML Patients treated using 7 + 3 + GO or FLAG-GO regimens to evaluate long-term OS and relapse rates.
- Randomized clinical trials comparing the safety and the effectiveness of 7 + 3 + GO vs. FLAG-GO.
- International harmonization for the methodology and the criteria of MDR assessment in t(8;21) and inv(16) AML patients.
- Definition of the criteria of risk stratification of CBF-AML patients through the integration of clinical parameters, mutational profiles and chromosome abnormalities.
- Definition of new criteria for the optimal timing of allo-HSCT, according to clinical and biological criteria and MDR evaluation, with consequent selection of patients for allo-HSCT at CR1 or at CR2.

Several studies have consistently shown the capacity of VEN in association with HMA to induce a high rate of CR in unfit or elderly CBF-AML patients and represent the optimal therapy for these patients. However, in spite of these high rates of responses, VEN-based regimens are associated in these patients with an OS shorter than that expected for favorable-risk patients, suggesting that the patients should be reclassified as intermediate-risk patients in ELN 2024. Future prospective studies will be required to carefully assess the response of elderly unfit CBF-AML patients to VEN-based regimens and to compare this response to that observed in elderly fit CBF-AML patients treated with IC.

According to all these considerations, a tentative algorithm for treatment of fit and unfit CBF-AML patients is proposed (Figure 4).

## Figures and Tables

**Figure 1:**
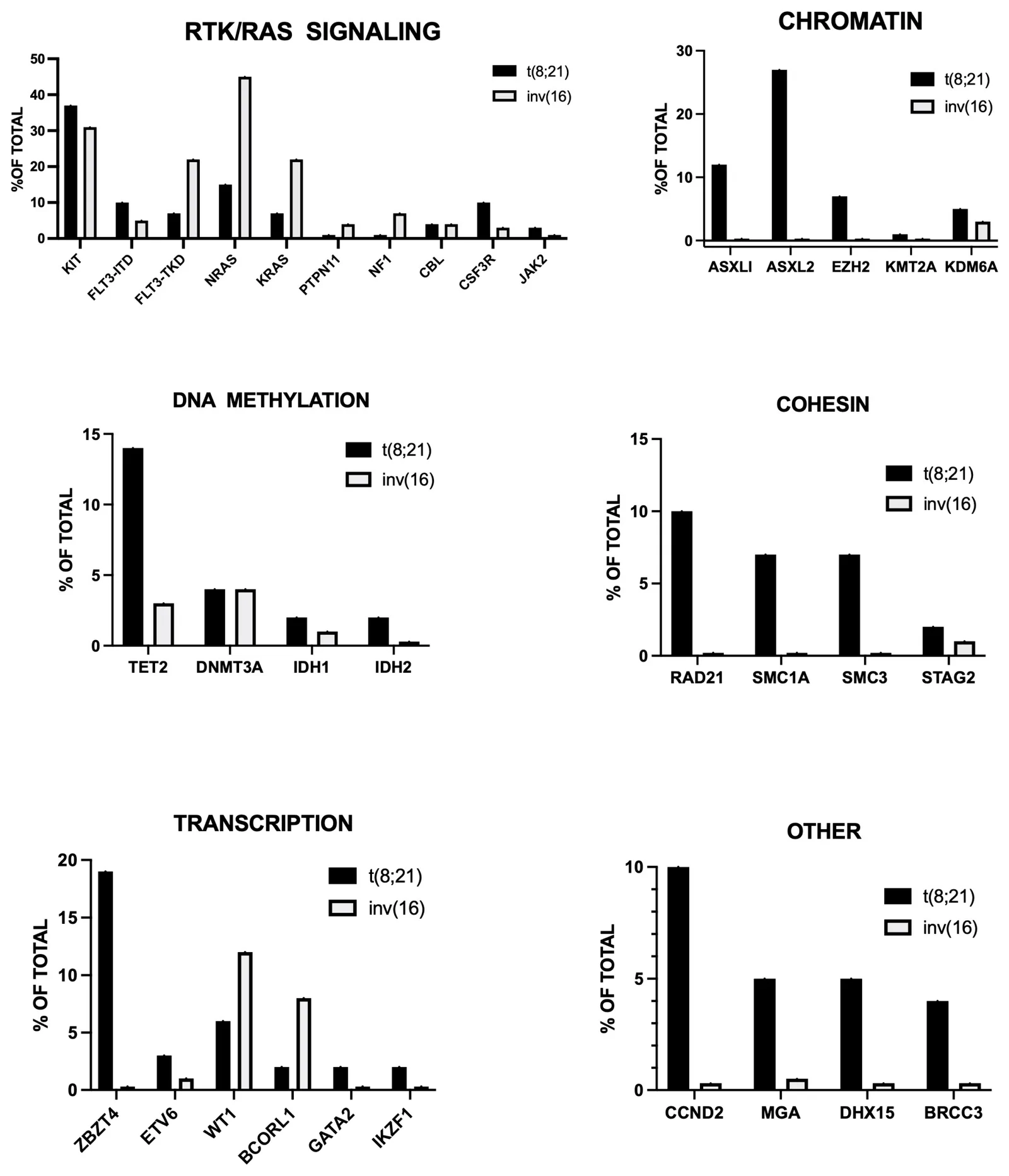
Most recurrent gene mutations observed in t(8;21) and inv(16) AMLs, subdivided according to different gene pathways. Original data were reported by Faber et al., 2016 [19].

**Figure 2:**
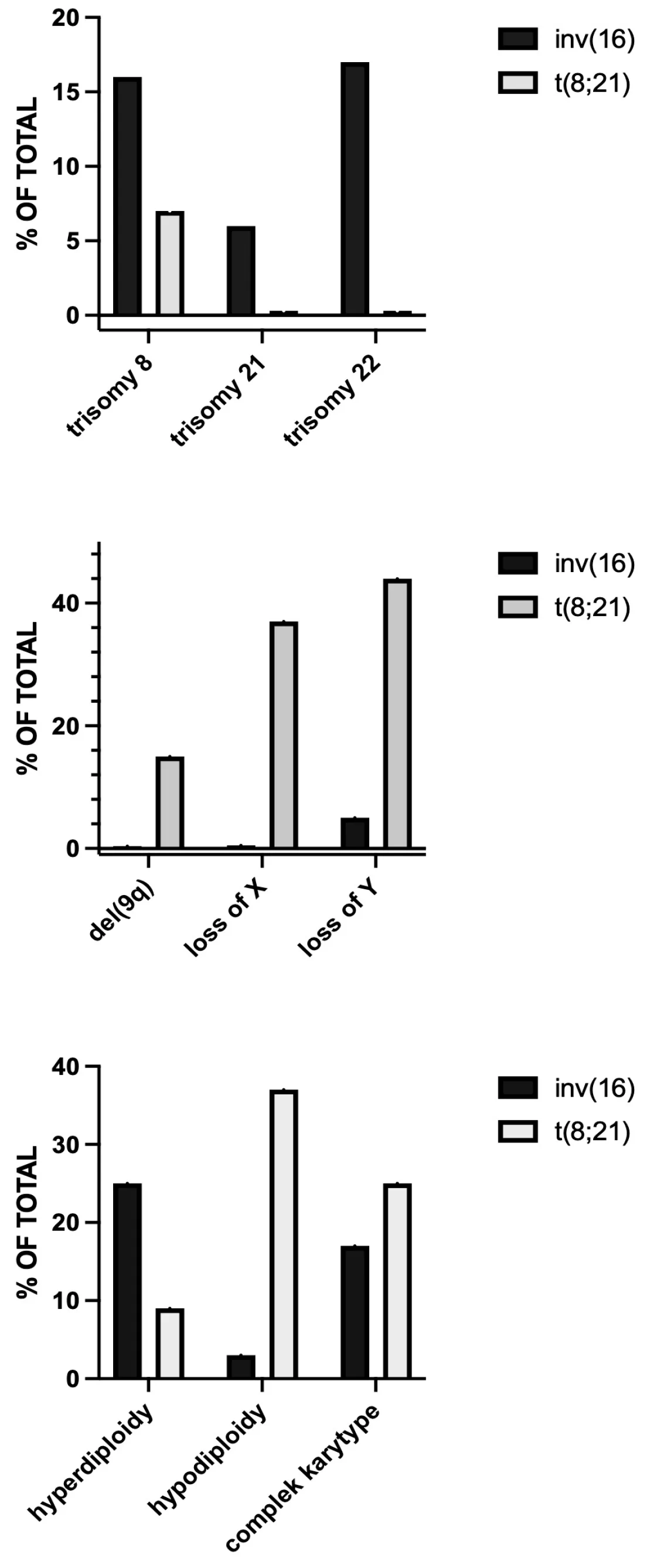
Most recurrent chromosomic alterations observed in t(8;21) and inv(16) AML. Original data were reported in Han et al., 2021 [24].

**Figure 3:**
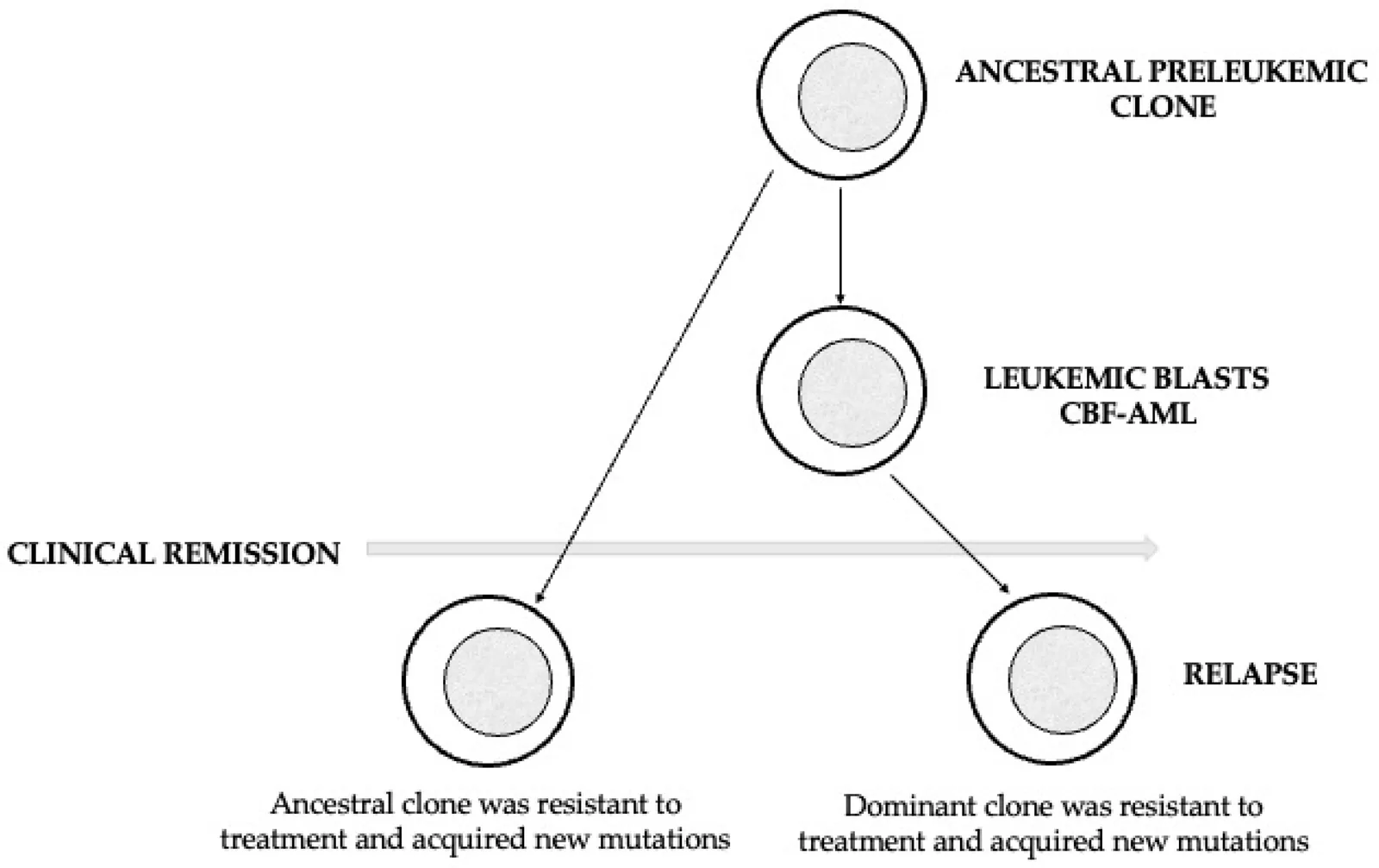
Model of clonal evolution leading to relapse, as proposed by Sood et al. [31]. According to this model, relapse in CBF-AML may originate from the acquisition of new mutations either in a dominant leukemic clone resistant to induction and consolidation treatments or in an ancestral preleukemic clone resistant to induction and consolidation treatments.

**Figure 4:**
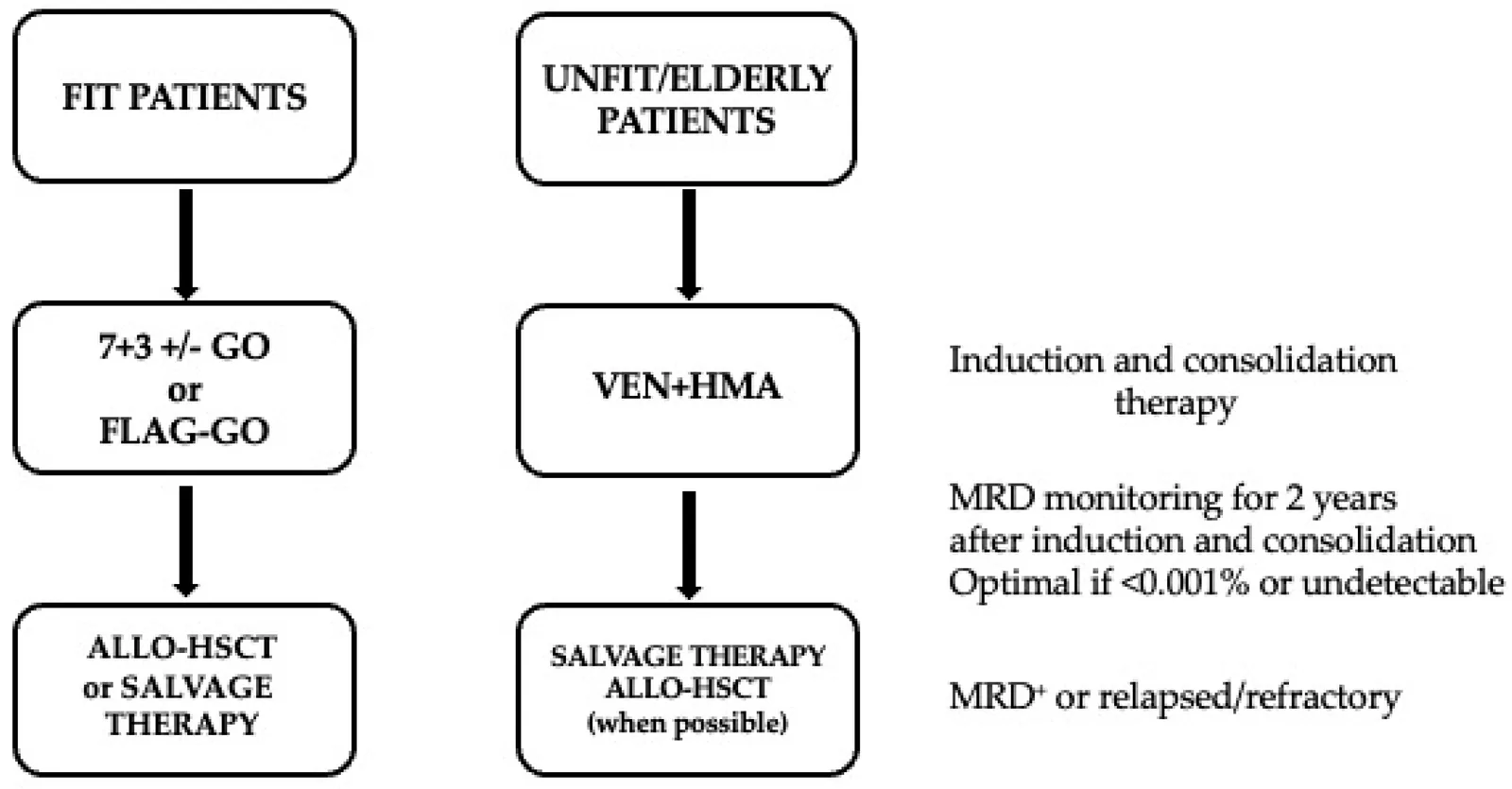
Treatment algorithm for fit and unfit/elderly CBF-AML patients.

**Table 1:** Frequency and prognostic impact of the main secondary mutations observed in t(8;21) and inv(16) AMLs.

Gene Mutation	Frequency in CBF-AML	Prognostic Impact in t(8;21) AML	Prognostic Impact in inv(16) AML
KIT (exon 17 D816, D820, N822) KIT (exon 8)	t(8;21) *KIT^Exon17^* 25–27%; *KIT^Exon8^* 12–15% inv(16) *KIT^Exon17^* 12–15%; *KIT^Exon8^* 18–22%	Adverse for exon 17 mutations but not for exon 8 mutations. Adverse for *KIT*-mutant high allelic ratio. Guide for allo-HSCT in CR1	Not consistent adverse impact
FLT3-ITD FLT3-TKD	t(8;21) *FLT3-ITD* 7–8%; *FLT3-TKD* 3.5–4.5% inv(16) *FLT3-ITD* 4–5%; *FLT3-TKD* 13–15%	Negative impact on outcome for *FLT3-ITD* but not for *FLT3-TKD*	Negative impact on outcome for *FLT3-ITD*, except those with trisomy 22
NRAS KRAS	*NRAS* mutations more frequent than *KRAS* mutations t(8;21) 25–30% inv(16) 35–55%	The prognostic impact is unclear. With some induction regimen the prognostic impact is favorable.	The prognostic impact is unclear. In pediatric patients the prognostic impact is favorable
ASXL2	15–20% t(8;21) AML	No significant impact on relapse/survival.	Rare/absent
ZBTB4	10–20% t(8;21) AML Missense and truncating mutations in the C-terminal zinc-finger Domain	Implicated in leukemogenesis. No significant impact on relapse/survival.	Rare/absent
Cohesin Complex	20–25% in t(8;21) AML *RAD21*, *SMC3*, *SMC1A*, *STAG2*	Implicated in leukemogenesis. Clinical prognosis unclear; in some studies cohesin complex mutations or *SMC1A* mutations favored poorer survival/relapse.	Rare/absent

**Table 2:** Current induction chemotherapy regimens for CBF-AML patients.

Chemotherapy Regimen	7 + 3 + GO	FLAG-GO	FLAG-IDA
Induction	Cytarabine × 7 days Daunorubicin or Idarubicin × 3 days Gemtuzumab Ozogamicin	Fludarabine	Fludarabine
		Cytarabine	Cytarabine
		G-CSF	G-CSF
		Gemtuzumab Ozogamicin	Idarubicin
Consolidation	High-dose Cytarabine	FLAG-GO	FLAG-IDA
Clinical efficacy	+GO vs. −GO	CR+CRi 92%	CR+CRi 85%
	CR+CRi 89% vs. 90%	5-yr OS 80.1%	5-yr OS 69.5%
	2-yr OS 90% vs. 80%	5-yr RFS 79.5%	5-yr RFS 59.4%
	2-yr EFS 51% vs. 36%	Rel Rate 17%	Rel Rate 28%
	2-yr CIR 42% vs. 50%	MDR^neg^ 92%	MDR^neg^ 85%
	MDR^neg^ 77% vs. 49%	OPR 72.8%	OPR 29.4%

The table reports the results observed for 7 + 3 + GO treatment in the study of Ronnacker et al., 2025 [91], and the results observed in the study of Senapati et al., 2026 [92], for FLAG-GO and FLAG-IDA.

## Data Availability

No new data were created or analyzed in this study. Data sharing is not applicable to this article.
